# Conformational Switching Controls Biradical Spin Dynamics in Flavin–Tryptophan Dyads

**DOI:** 10.1021/jacs.5c22947

**Published:** 2026-03-23

**Authors:** Guzel Musabirova, Olga B. Morozova, Alexey S. Kiryutin, Irina S. Anisimova, Ivan V. Zhukov, Tobias Theiss, Luca Gerhards, Ben G. E. Zoller, Tanja Gulder, Jörg Matysik, Ilia A. Solov’yov, Alexandra V. Yurkovskaya

**Affiliations:** † Department of Analytical Chemistry, Leipzig University, Linnéstr. 3, 04103 Leipzig, Germany; ‡ Siberian Branch of the Russian Academy of Science, International Tomography Center, Institutskaya 3A, 630090 Novosibirsk, Russia; § Institute of Physics, Carl von Ossietzky Universität, Carl-von-Ossietzky-Street 9−11, 26129 Oldenburg, Germany; ∥ Department of Organic Chemistry, Leipzig University, Johannisallee 29, 04103 Leipzig, Germany; ⊥ Organic Chemistry, 9379 Saarland University, 66123 Saarbrücken, Germany; # Research Center Neurosensory Science, Carl von Ossietzky-Universität, Carl-von-Ossietzky-Street 9−11, 26129 Oldenburg, Germany; ¶ Synthesis of Natural-Product Derived Drugs, Helmholtz Institute for Pharmaceutical Research Saarland (HIPS) Helmholtz Centre for Infection Research (HZI), 66123 Saarbrücken, Germany; ∇ Center for Nanoscale Dynamics (CENAD), Carl von Ossietzky-Universität, Carl-von-Ossietzky-Street 9−11, 26129 Oldenburg, Germany

## Abstract

Flavin–tryptophan dyads linked by oligoproline chains were used to probe how proline *cis*–*trans* isomerization modulates spin dynamics under photo-CIDNP conditions. Field-cycling NMR revealed that minor *cis*–containing conformers form compact geometries that support intramolecular biradical recombination. These states display the features of *J*—resonance photo-CIDNP: uniform emissive polarization and pronounced maxima at 5–20 mT, while their signals vanish at high fields. In contrast, the dominant all-*trans* (PPII) conformers and longer linkers produce only Δ*g*—dominated patterns consistent with intermolecular radical encounters. Time-resolved photo-CIDNP confirmed that intermolecular processes prevail in most systems, with the four-proline dyad showing additional intramolecular contributions. The results establish that proline isomerization serves as a structural switch that governs the donor–acceptor distance and, consequently, the balance between intra- and intermolecular spin-selective pathways. The study further highlights field-cycling photo-CIDNP as a promising tool for identifying transient biradical states in biomolecular model systems.

## Graphical abstract

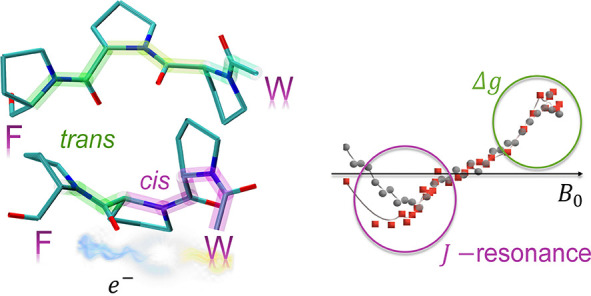

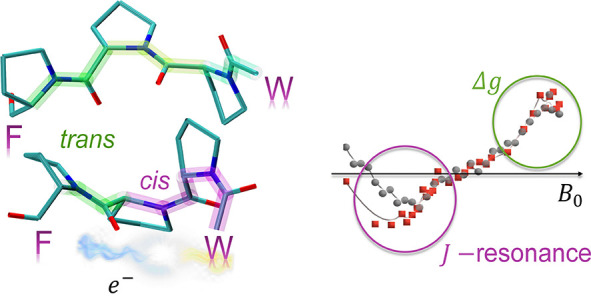

## Introduction

Flavoproteins are widespread in nature and serve as key components in biological blue-light photoreception. A representative example here are the light-oxygen-voltage (LOV) domains.^1–6^ Upon photoexcitation, these systems form short-lived radical pairs that can give rise to spin-correlated phenomena such as photochemically induced dynamic nuclear polarization (photo-CIDNP).^7–13^ Understanding the spin dynamics in such systems offers insight into biological signaling, electron transfer, and the structural organization of proteins.

Nuclear magnetic resonance (NMR) is a noninvasive cornerstone technique for elucidating molecular structure, dynamics, and interactions in chemistry, material sciences, and biophysics.^6,13–17^ Although NMR is widely used in structural biology, and magnetic resonance imaging (MRI) has become an indispensable diagnostic tool in human medicine, NMR suffers from inherently low sensitivity due to the small population differences between nuclear spin states, which are governed by Boltzmann statistics.^18,19^ Hyperpolarization techniques overcome this limitation by generating transient, nonequilibrium spin populations that enhance NMR signals by several orders of magnitude.^20–22^

Among various hyperpolarization strategies, photo-CIDNP utilizes light-triggered chemical reactions (e.g., electron transfer, bond cleavage, etc.) to create spin-correlated radical pairs (SCRPs), having high spin-order, which can be transferred to nuclei through electron–electron-nuclear spin dynamics.^1,23^ In liquid-state systems, this effect is often interpreted within the framework of the classical radical-pair mechanism (RPM),^24–26^ although its direct applicability to covalently linked donor–acceptor systems is limited.

To explain polarization behavior beyond the standard RPM, several theoretical extensions have been proposed. In particular, field-dependent polarization patterns have been rationalized through the concepts of level crossings (LC) and level anticrossings (LAC).^23,27^ These phenomena describe the conditions under which energy levels of the spin system approach or avoid each other as a function of the external magnetic field. At an LAC, mixing between spin states is most efficient, resulting in enhanced nuclear polarization. The emergence of LACs depend on molecular parameters such as the exchange interaction *J*_ex_, hyperfine coupling *a*, and *g*-factor differences Δ*g*, for example described recently.^23^ The sign of the polarization Γ in liquid-state photo-CIDNP is determined by the interplay between these parameters, the precursor multiplicity, and the relative probability of the recombination of spin-correlated geminate and uncorrelated radical pairs formed from escape radicals.^24,25^ Table 1 summarizes these sign rules.

**Table 1:** Sign Rules for CIDNP Polarization (Γ) in Spin Correlated Radical Pairs under Different Magnetic Field Regimes^a^

mechanisms	sign rules
Δ*g*—dominated regime (high magnetic field)	Γ = sgn(Δ*g*) × sgn(*a*) × μ × ε
Electronic exchange-dominated regime (limited distances, at the LAC between the singlet S and triplet T_+_ or T___ states)	Γ = sgn(J_ex_) × μ × ε

a Here μ = +1 for triplet precursors and μ = −1 for singlet precursors, ε = +1 for geminate recombination and ε = −1 for escape products.

In solid-state systems (for review, see^11^) photo-CIDNP has been predominantly observed in photosynthetic reaction centers^28,29^ and protein-bound flavins, particularly in LOV domains.^30,31^ In contrast, organic-synthetic systems, such as the artificial dyads and rigid bichromophoric conjugates, have demonstrated this effect mainly in solution.^32–36^ While protein scaffolds provide constrained donor–acceptor geometries, their structural complexity limits experimental structural tunability. In contrast, synthetic conjugates provide a modular and tunable platform for field-dependent spin studies, allowing for systematic control over distance, orientation, and conformational degrees of freedom. Recently, De Biasi et al. demonstrated the occurrence of a solid-state photo-CIDNP effect in stiff molecular scaffolds.^37^

Field-dependent photo-CIDNP experiments have elucidated the interplay of several factors, including the spin selectivity in radical reactions, the conservation of total electron and nuclear spins, and their hyperfine interactions. These experiments have demonstrated a correlation between molecular geometry and exchange interactions.^33,38–42^ Nonetheless, the influence of molecular conformational dynamics on field-dependent photo-CIDNP has not been thoroughly explored.

In this context, molecular systems comprising flavin and tryptophan moieties connected by polyproline linkers represent an attractive and well-defined platform to investigate the influence of conformational dynamics on spin-dependent processes.^43^ Polyproline chains are known to adopt two distinct helical conformations: the polyproline I (PPI) helix, characterized by predominantly *cis* amide bonds, and the polyproline II (PPII) helix, in which the amide bonds adopt the *trans* configuration.^44^ The relative stability and population of these helices depend sensitively on the number of proline residues, solvent polarity, temperature, and specific intramolecular interactions.^45^ Importantly, *cis*–*trans* isomerization of the peptidyl–prolyl amide bond is intrinsically slow on the NMR time scale (exchange rates on the order of 10^–2^–10^–1^ s^–1^), giving rise to long-lived conformational heterogeneity in oligoproline chains.^46^

Previous studies have shown that flavin-tryptophan dyads bridged by polyproline linkers exhibit steady-state ^1^H photo-CIDNP effects in solution.^43^ In that work, we have demonstrated that the fraction of PPII helices strongly increases with linker length, ranging from negligible for three proline units to approximately 70% for 12-proline chains, while a small but non-negligible population of *cis*-containing conformers persists even for longer linkers. Although such *cis* defects are often regarded as structural imperfections, they introduce local bends and fluctuations in the linker geometry that can substantially modify donor–acceptor distances, electronic coupling, spin-exchange interactions, and radical-pair lifetimes. Thus, polyproline-linked flavin-tryptophan dyads provide a simplified yet powerful model system in which conformational heterogeneity and its impact on spin-dependent reaction pathways can be isolated and systematically explored, features that are characteristic of many flexible redox-active molecular and biomolecular systems.

In our recent study on flavin-tryptophan oligoproline dyads,^43^ the dependence of steady-state high-field photo-CIDNP intensity on linker length was interpreted mainly in terms of a structure–activity relationship, with the six-proline linker emerging as an apparently optimal architecture for efficient hyperpolarization. That analysis, however, did not allow a clear separation between intra- and intermolecular radical-pair pathways. The present results substantially clarify this interpretation. Time-resolved photo-CIDNP kinetics at high field demonstrate that, for the major all-*trans* (PPII) conformers of the dyads, CIDNP is generated predominantly via bimolecular encounters of flavin- and tryptophan-centered radicals in solution. In contrast, field-cycling experiments reveal that only minor *cis*-containing conformers of short oligoproline linkers exhibit pronounced low- and intermediate-field features characteristic of exchange-mediated (*J*-dominated) spin evolution, consistent with intramolecular biradical recombination in compact geometries. Thus, the strong photo-CIDNP effects previously observed for the six-proline dyad should not be interpreted as evidence for a predominantly intramolecular mechanism; rather, they mainly reflect efficient intermolecular radical-pair formation, while intramolecular CIDNP is confined to a small subpopulation of conformers. This revised picture establishes proline *cis*–*trans* isomerization as a structural switch that governs access to intramolecular spin-correlated radical pairs and highlights the necessity of field-dependent and time-resolved approaches for reliable mechanistic assignment in covalently linked donor–acceptor systems.

The present study investigates the magnetic-field dependence of nuclear hyperpolarization in flavin-tryptophan (F–W) oligoproline dyads using ^1^H liquid-state field-cycling NMR across magnetic fields ranging from 1 mT to 9.4 T. Remarkably, shorter dyads (containing 3 and 4 prolines) exhibit pronounced variations in CIDNP signal intensity that cannot be explained solely by distance-dependent exchange interactions. This observation suggests that additional structural factors, in particular *cis*–*trans* isomerization of the amide bond in proline residues, play a role.

To comprehensively address the problem, the study combines field-cycling and time-resolved photo-CIDNP NMR with nuclear spin relaxation, diffusion measurements, molecular dynamics simulations, and quantum chemistry calculations.^33,47–49^ The synergy of the methods allowed us to probe both the spin dynamics and conformational behavior of the dyads. Our findings reveal that conformational heterogeneity of the polyproline linker controls both the efficiency and the dominant mechanism of nuclear spin polarization. By combining field-cycling photo-CIDNP with complementary spectroscopic and computational approaches, this work demonstrates how conformational heterogeneity governs spin-selective reactivity and highlights the potential of field-dependent CIDNP to probe transient biradical states in flexible molecular systems.

## Materials and Methods

### General Considerations

W–Pro_*n*_–F dyads (*n* = 3–12) were synthesized as reported previously.^43^ N10-(4-carboxyphenyl)-flavin was used as a reference. Deuterated solvent (methanol-*d*_4_, 99.8%) purchased from Carl Roth was used as received.

### Sample Preparation

Solutions were prepared in MeOD-*d*_4_ at OD ≈2 at 450 nm (1 cm). Samples were degassed by bubbling argon for 6–10 min before the measurements.

### UV–Vis Absorption

UV–Vis spectra (200–600 nm, 1 nm resolution) were recorded in 0.5 cm path length quartz cells with baseline correction against solvent. Fresh, minimally photobleached samples were used to obtain final data sets.

### NMR Spectroscopy (General)

1D ^1^H spectra were acquired at 600 MHz (Bruker, 5 mm TBI probe) at 293 K using standard pulse programs. 2D ^1^H–^13^C HSQC (hsqcetgp) spectra were measured on a Bruker Avance III HD 700 MHz spectrometer using typical parameters of 4096 × 512 complex points, d_1_ = 3 s, and 32 scans per increment. Diffusion measurements employed BPP-STE (ledbpgp2s) with linearly incremented gradients (2–95% of 50 G cm^–1^), Δ = 50–100 ms, δ = 2–4 ms, and d_1_ = 2 s; DOSY analysis was performed via monoexponential fitting of signal decay (Stejskal–Tanner model). Data were processed in TopSpin 4.0.9 and MestReNova 14.2.1.

Note. Further details of field-cycling, time-resolved photo-CIDNP experiments and relaxometry details (instrument models, laser/LED power at the sample, shuttle kinematics, and complete parameter tables) are provided in the Supporting Information.

### Computational Methods

Conformational ensembles were generated with CREST;^50,51^ representative structures were refined, and the chemical shielding was computed in ORCA 6.0 ^52,53^ (TPSSh/EPR-II, CPCM/MeOH). Classical molecular dynamics (MD) simulations employed NAMD, following ref;^43^ production totaled 1.44 μs per proline conformation. Full set of simulation settings are provided in the Supporting Information.

## Results

Molecules consistent of a flavin (F) chromophore acting as an electron acceptor and a tryptophan (W) residue serving as an electron donor, connected by an oligoproline linker of variable length (*n* = 3–12) (see Figure 1). The polyproline linker is conformationally restricted, allowing for predictable control of the donor–acceptor distance. This enables a systematic investigation of the effect of distance and linker conformation on the photophysical and spin dynamic properties of the dyads.

**Figure 1:**
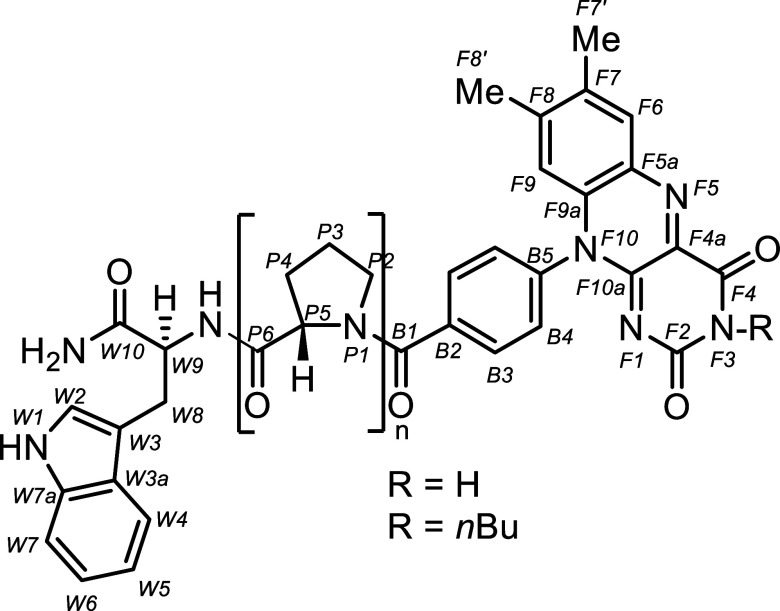
General structure of the studied W–Pro_*n*_–F = *n*Pro dyads. The dyads consist of a tryptophan moiety acting as an electron donor (W), a polyproline spacer (Pro, *n* = 3–12), and a flavin acceptor unit (F). Two series of dyads were studied: R = H with Pro units *n* = 3, 4, 6, 9, 12, and R = *n*Bu with Pro units *n* = 3, 6, 9, resulting in eight dyads in total.

### Observation of Distinct Conformations by NMR

One-dimensional ^1^H NMR spectra of the F–W conjugates with 3, 4, 6, 9, and 12 prolines revealed clear differences in both the aromatic region (6.5–8.5 ppm), where signals from the F and W moieties appear, and methyl region (2.35–2.50 ppm) corresponding to the two methyl groups F7′ and F8′ of the flavin chromophore (see Figure 2; chemical shift information introduced in Table S1). The remaining parts of the spectra, can mostly be attributed to proline resonances and are dominated by broad, overlapping signals, which limit resolution and prevent detailed analysis of individual peaks in this region.

**Figure 2:**
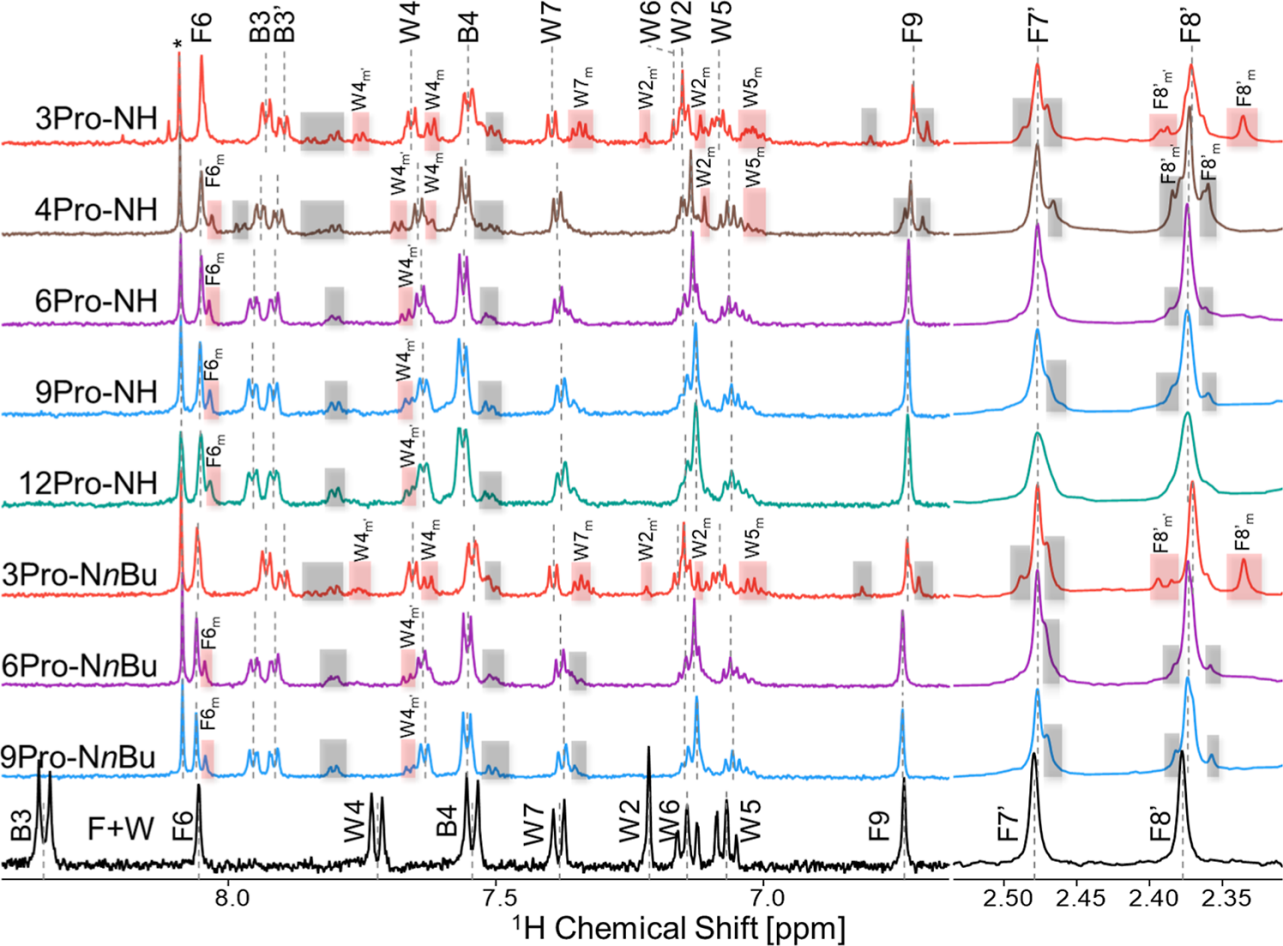
^1^H NMR spectra (600 MHz, MeOD-*d*_4_, 293 K) recorded for the F–W dyads (10 mM) with oligoproline linkers. Spectra are shown for two series of dyads: –NH series (top five traces) and -N*n*Bu series (further three traces). The spectrum of N10-(4-carboxyphenyl)-flavin and W without a linker is shown in black at the bottom and is marked as F + W. Vertical dashed lines indicate the assigned signals of the major conformer, labeled at the top (e.g., F6, B3, W6). Minor conformer signals are labeled with an additional m subscript (e.g., W6_m_) and appear outside the dashed regions. Red highlights mark the minor signals that exhibit photo-CIDNP enhancement, indicating spin polarization in a subpopulation of conformers. Minor signals without observable CIDNP effect are shaded in gray. A progressive reduction in the number and intensity of minor conformer signals is observed with increasing linker length, indicating a decrease in conformational heterogeneity. The N10-(4-carboxyphenyl)-flavin and W signals are labeled separately, as their chemical shifts differ significantly from those in the oligoproline-linked dyads. The asterisk (*) denotes an impurity-related peak.

The most significant number of minor signals (W4_m′_, W4_m_, W7_m_, W2_m′_, W2_m_, W5_m_, F8′_m′_, F8′_m_) was observed for the 3Pro dyads, with similar patterns for both –NH and -N*n*Bu series. The 4Pro dyads still displayed numerous minor peaks, comparable to the 3Pro case, although some signals (e.g., W7_m_) disappeared, others became shifted (e.g., W4_m′_), and a new minor signal near the F6 signal (labeled F6_m_) appeared. The F6_m_ signal was observed in the NMR spectrum for all dyads with 4 or more prolines. In the 6, 9, and 12 proline dyads, the number of minor peaks is minimal (F6_m_, W6_m′_), and the spectra appeared to be more homogeneous. This behavior reflects the increasing conformational rigidity of the proline linker and a reduction in structural heterogeneity with the length of the dyads, as confirmed by circular dichroism (CD) and 2D-^1^H NMR spectroscopy of these dyads.^43^ For comparison, the spectrum of only the F and W moieties, not connected by a polyproline linker (see Figure 2, bottom trace), shows no evidence of minor conformer signals, supporting the conclusion that the observed structural heterogeneity of the spectra recorded for the dyads originates from the oligoproline linker conformations.

A series of 2D ^1^H–^13^C HSQC spectra was recorded for selected dyads and is presented in the Supporting Information. For most protons, multiple signals were observed, which are more clearly resolved in the 2D spectra. For example, in the aromatic region of the 3Pro-N*n*Bu dyad, a small shoulder for the F9 proton is evident in HSQC but not visible in the corresponding 1D spectrum (see Figure S8). The W2 and W4 protons show at least three distinct cross-peaks each (see Figure S7). In the 6Pro-N*n*Bu dyad, only two minor signals are apparent in the 1D ^1^H spectrum; however, the HSQC spectra reveal additional conformations (see Figure S9). This is particularly evident for protons such as F9, W7, and W2, where each exhibits three or more cross-peaks, indicating the presence of multiple slowly exchanging conformers.

### Time-Resolved CIDNP Measurements

Time-resolved (TR) photo-CIDNP experiments were performed to investigate the mechanism of CIDNP formation in solution at a high magnetic fields. TR photo-CIDNP spectra obtained during irradiation of the conjugate solution immediately after the laser pulse are shown in Figure S14. The CIDNP pattern is typical for that of nonconjugated W and F moieties and follows Kaptein’s rule for CIDNP signs in the context of the RPM mechanism.

To distinguish whether CIDNP originates from intramolecular electron transfer via biradical formation or from intermolecular reactions between flavin- and tryptophan-centered monoradicals of different conjugates, TR photo-CIDNP kinetics were examined in detail for 0.2 mM 3Pro-N*n*Bu in MeOD. CIDNP spectra were acquired using a 2 μs detection pulse at ten fixed delays after the laser flash, ranging from 0 to 100 μs (see Figure S15). The 3Pro-N*n*Bu dyad was selected for this detailed analysis because it exhibits the highest photostability and the most reproducible CIDNP response under continuous irradiation among all investigated systems. The resulting kinetic traces display temporal behavior characteristic of an intermolecular polarization mechanism. On this basis, TR photo-CIDNP experiments for the remaining dyads were performed using three representative delay times (0, 3, and 100 μs), which capture the initial buildup, near-maximum intensity, and long-time decay of the CIDNP signal.

In these experiments, the duration of the detection RF pulse was 4 μs. In a first approximation, the effective time of the acquired spectra can therefore be taken as the center of the detection pulse, corresponding to 2 μs, 5 μs, and 102 μs after the laser flash. The limited number of time points was dictated by the restricted amount of sample available and by photodegradation of several dyads under laser irradiation, which precluded accumulation of a larger number of spectra with sufficient signal-to-noise ratio for reliable analysis.

For the kinetic analysis, signal intensities of the tryptophan protons W2 and W6, and the flavin proton F8′ were evaluated. The corresponding photo-CIDNP kinetics are shown in Figure 3. The signal of tryptophan W8 protons overlaps with residual methanol resonances that are not completely suppressed by the homonuclear decoupling pulses. Consequently, despite its higher intensity, the W8 proton signal was unsuitable for quantitative analysis. For dyads containing three prolines, the polarization of both W and F increased at 5 μs compared to a measurement at 2 μs. The CIDNP kinetics of the donor exhibits a distinct maximum in all conjugates. For the flavin moiety, a pronounced maximum is observed for the 3Pro-N*n*Bu, 3Pro-NH, and 6Pro-NH dyads, whereas in the 9Pro-NH and 12Pro-NH dyads, the polarization increased monotonically without reaching a maximum within the investigated time window.

**Figure 3:**
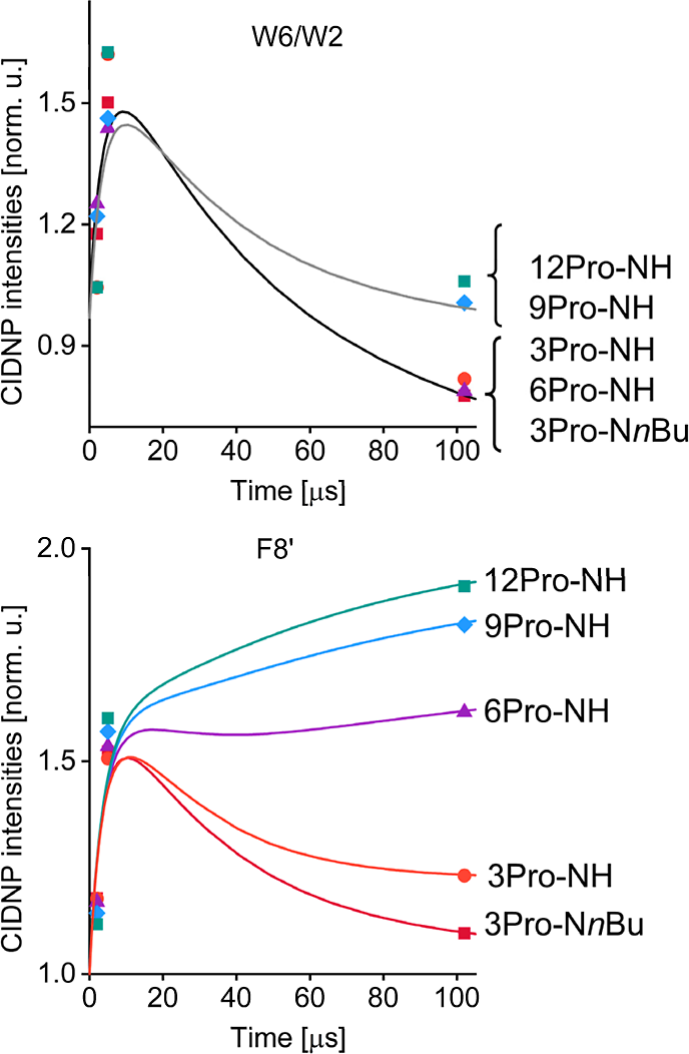
Time-resolved photo-CIDNP kinetics of F–W dyads with proline linkers of varying length (3–12 residues) measured at 4.7 T and 293 K, 0.2 mM in MeOD-*d*_4_. Time refers to the center of the detection RF pulse (4 μs duration) applied after 20 ns pulsed laser excitation at 308 nm. Dots: experimental data (top, W2/W6 protons; bottom, F8′ protons). Lines: simulations using Fisher’s cyclic photoreaction model for the bimolecular triplet quenching mechanism.

In general, CIDNP kinetics fully corresponds to behavior characteristic of cyclic photoreactions involving radical pairs as intermediates. In such systems, radicals are generated through bimolecular triplet quenching, and the resulting pair of two monoradicals subsequently decays in second-order reactions.

Equations proposed by Fischer et al.^54^ for the CIDNP kinetics in cyclic photoreactions were used to simulate CIDNP kinetics. These equations include the concentration of radicals, *R* (dye radicals or quencher radicals), the polarization of the nucleus under consideration in radicals, *P*_R_, and in recombination products, *P*

1 $$R\left(t\right)=\frac{R_{0}}{1+k_{t}R_{0}t}$$

2 $$\frac{dP_{R}}{dt}=-k_{t}P_{R}R-k_{t}\beta R^{2}-\frac{P_{R}}{T_{1}}$$

3 $$\frac{dP}{dt}=k_{t}P_{R}R+k_{t}\beta R^{2}$$

here, *k*_*t*_ is the rate constant of the second-order termination reaction between F radicals and W radicals; *T*_1_ is the paramagnetic relaxation time of the nucleus under consideration. The parameter β represents the polarization per pair created upon recombination of F-pairs during encounters of radicals in the bulk. It is related to the geminate polarization *P*_G_ via the parameter γ, defined as the ratio of polarization created in the F-pairs to the geminate polarization: β = γ*P*_G_/*R*_0_; here *R*_0_ is the initial concentration of radicals of each type that escaped geminate recombination and are involved in F-pair reactions. In the case of a triplet precursor γ = 3. We have, however, assumed γ = 2.8 to account for the incomplete reversibility of the photoreaction, as suggested by Fischer.^54^ *T*_1_, *k*_*t*_ *R*_0_, and the vertical scaling factor were thus the fitting parameters used in the simulations.

Parameters *k*_*t*_ *R*_0_ for the different dyads were determined from kinetic fitting of the CIDNP time profiles to be close to 10^5^ s^–1^ (see Table 2), and were assumed to be the same for all conjugates; these parameters were then used for the calculations of the profiles of the time-resolved kinetics shown in Figure 3. Following the value of *T*_1_, the kinetics for W were divided into two groups, corresponding to *T*_1_ ≈ 500 μs for 3Pro-N*n*Bu, 3Pro-NH, 6Pro-NH, and *T*_1_ ≈ 150 μs for 9Pro-NH, 12Pro-NH. Not that these *T*_1_ values are much longer than those found for W2, W6 of *N*-acetyl tryptophan radical (44 μs).^55^ For the Flavin moiety, whose signal is higher, and the values of *T*_1_ better matches the ‘kinetic window’ of the CIDNP experiment, all kinetics diverged in accordance with the values of *T*_1_ (see Table 2). In general, shortening of *T*_1_ upon increasing the number of prolines reflects an increase in rotational correlation time.

**Table 2:** Parameters Obtained from the Simulation of CIDNP Kinetics: Nuclear Paramagnetic Relaxation Times *T*_1_ and Characteristic Time of Second-Order Radical Decay *k*_*t*_ *R*_0_

	*T*_1_, μs		
	F8′	W6/W2	*k*_*t*_ *R*_0_, s^–1^
3Pro-N*n*Bu	120	500	1 × 10^5^
3Pro-NH	77		
6Pro-NH	38		
9Pro-NH	27	150	
12Pro-NH	23		
4Pro-NH	19	29	4.2 × 10^5^

For the 3Pro-N*n*Bu conjugate, which exhibited the highest photostability among the samples, CIDNP kinetics were also measured at 10 time points using a 2 μs detection pulse (see Figure S15). The scatter of points was larger, but overall, the kinetics corresponds to what was obtained for three time points using a 4 μs detection pulse, discussed above.

The results indicate that if biradicals are formed in W-(Pro)_n_-FNH (*n* = 3, 6, 9, 12) and W-(Pro)­3-FN*n*Bu in MeOD-*d*_4_ systems, their contribution to the observed kinetics at a high magnetic field of 4.7 T is insignificant and the detected CIDNP signals under laser-pulsed irradiation are formed exclusively through intermolecular electron transfer.

The case of 4Pro-NH stands apart. For this dyad, CIDNP decays monotonously (see Figure S16). The pseudo-first order decay of radicals, as described above, is 4 times higher and the relaxation times are shorter than for other dyads (see Table 2); *T*_1_ of W6/W2 is particularly significantly shortened. Considering the scatter of experimental data and the low time resolution associated with using a 4 μs pulse, these observations can be explained by a relatively slow monomolecular reaction in the biradical, with the lifetime of the latter being approximately 5–10 μs.

### Magnetic Field Dependence of Photo-CIDNP

Here, we focus on how isomerization-driven heterogeneity in short linkers contributes to the observed spin dynamics under photo-CIDNP conditions. To understand the role of the linker length, we have systematically measured field-dependent photo-CIDNP NMR effects for each dyad in the series (see Figure 4), as well as for the unlinked reference F–W system. Field-cycling experiments were performed across a wide range of magnetic fields, spanning from 1 mT to 9.4 T, with 30 evenly spaced field points on a logarithmic scale to ensure detailed sampling of both low- and high-field regimes (results for the –NH dyads are shown in Figure 4, for the -N*n*Bu dyads see Figure S17). Interpretation of the field dependences is complicated by the slight influence of the polyproline linker isomerization state on chemical shifts of F and W protons, which causes overlap of signals from different conformers.

**Figure 4:**
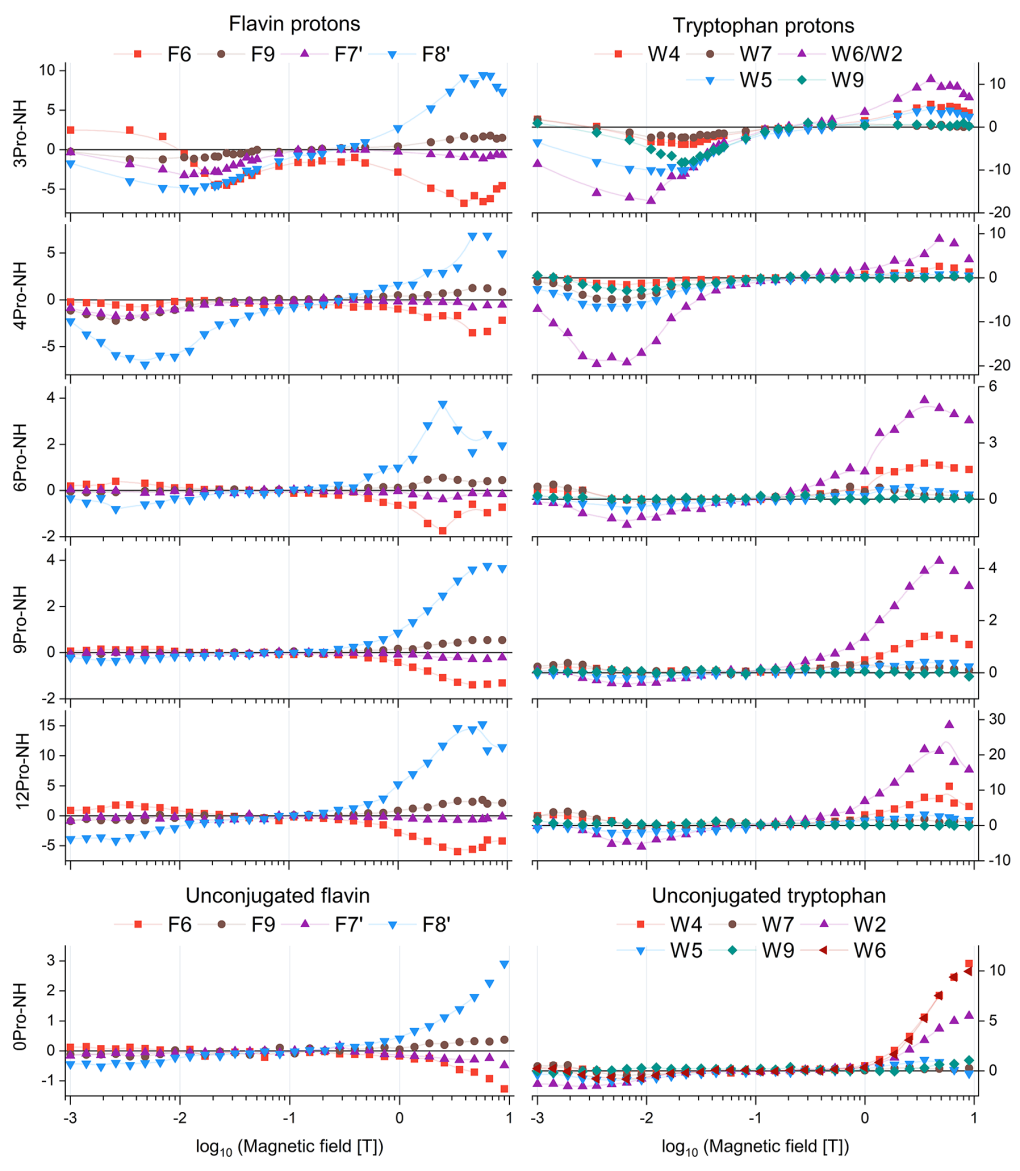
Magnetic field dependence of photo-CIDNP intensities for individual protons in F–W dyads and unconjugated references. Each curve shows the polarization amplitude of a specific proton signal as a function of magnetic field, measured after irradiation with a 450 nm LED. Left panels: flavin (acceptor) protons F6, F9, F7, and F8′; right panels: tryptophan (donor) protons W4, W7, W6/W2, W5, and W9. Rows correspond to different samples: 3Pro-NH, 4Pro-NH, 6Pro-NH, 9Pro-NH, 12Pro-NH, and unconjugated flavin and tryptophan. Data are plotted versus log_10_ of the magnetic field (*T*); lines are used for guidance.

All dyads and the unconjugated reference showed qualitatively similar CIDNP profiles in the high field (*B*_0_ ≥ 1 *T*) (see Figure S18). Polarization was restricted to signals of the major conformers. The strongest signal from the flavin was consistently stemming from the F8′ methyl group, whereas the F7′ methyl protons were contributing weakly with an opposite sign; additional polarization was detected for aromatic protons F6 and F9, also with opposite signs. On the tryptophan side, pronounced polarization was observed for W4 and W6/W2, while weaker signals from W9, W7, and W5 were detected in shorter dyads (*n* = 3, 4) and in the unconjugated reference. The observed polarization signs were consistent with the Kaptein rules^24^ and matched those of the unconjugated reference.

The overall polarization intensity decreased with increasing linker length, except for the 12Pro-NH dyad, which showed unexpectedly higher intensity. In addition, a systematic difference between –NH and -N*n*Bu derivatives becomes apparent for dyads with 6 and 9 prolines (see Figures S19–S21): in these cases, CIDNP intensities are consistently weaker for the –NH group compared to the -N*n*Bu group. This effect may be related to the higher photostability of the butyl-substituted derivatives, which could sustain radical pair generation more efficiently under prolonged irradiation. In the field range between 0.1 and 1 *T*, no appreciable CIDNP signals were detected for any proton (see Figure 4), indicating a magnetic field interval where neither Δ*g*—nor *J*—resonance mechanisms are effective.

In the intermediate-field regime, polarization strongly depended on the linker length. Once *g*β*B*_0_ ∼ |*J*_ex_| and *g*β*B*_0_ ≫ *a*, biradicals may still generate CIDNP via the *J*—resonance mechanism, provided that exchange interaction is non-negligible.^23,25,56,57^ This mechanism produces uniform negative (emissive) polarization across all observed protons. Such behavior was observed only for short linkers (3Pro, 4Pro), whereas longer dyads (6Pro, 9Pro, 12Pro) and the unconjugated system showed no detectable CIDNP. For 3Pro-NH and -N*n*Bu dyads, maximal emissive polarization occurred between 13 mT and 30 mT, with the strongest signals at F8′ and W6/W2. In 4Pro-NH, the maximum shifted to lower fields (2–8 mT), where F8′ and W6/W2 displayed higher intensity compared to the 3Pro dyads, while the remaining protons had comparable amplitudes.

At low magnetic field intensities, where *g*β*B*_0_ ∼ *a*, the sign of the photo-CIDNP effect typically reflects the sign of the hyperfine coupling constant.^23^ In this regime, polarization was observed for all measured protons in both F and W units (see Figure 4). Unlike the intermediate-field regime dominated by the *J*—resonance mechanism, where all proton signals display uniform emissive polarization, the low-field regime shows nucleus-specific behavior: the polarization sign differs between protons within the same molecule. This pattern is consistent across all dyads, independent of linker length, as well as for the unconjugated reference system.

Notably, the β-protons of tryptophan (W8′, W8″ at 3.3 ppm) exhibit field dependences distinct from other nuclei (see Figure S24). Their polarization remains pronounced over the entire field range and shows opposite signs for the two β-protons. Such behavior is characteristic of a strongly coupled spin system where the Larmor frequency difference is smaller or comparable to the scalar coupling constants. In this regime, the two W8 spins form a quasi-singlet state as^58–60^

4 $$|S\rangle =\frac{1}{\sqrt{2}}\left(|\alpha \beta \rangle -|\beta \alpha \rangle \right)$$

which is protected from the dipole–dipole relaxation due to its antisymmetric nature. Consequently, the W8 spin pair displays significantly prolonged lifetimes,^61,62^ leading to enhanced and distinct CIDNP features compared to other protons. Importantly, this effect is consistently observed for all dyads and for the unconjugated system, suggesting that it originates from intrinsic properties of the W moiety rather than linker-dependent effects.

The influence of molecular structure on the *J*—resonance behavior was studied through the comparison of measurements for the NH- and N*n*Bu -substituted 3-proline dyads (see Figures S22 and S23). As shown in Figure 5, field-dependent CIDNP profiles are broadly similar between the two derivatives across all monitored protons (see Figure S19). However, pronounced differences are observed between major and minor conformers. The minor conformer signals W4_m′_ and W4_m_, W7_m_, W6_m_, W2_m_, W5_m_, F8′_m′_, F8′_m_ display intense polarization in the intermediate-field regime (2–30 mT), consistent with efficient CIDNP generation via the *J*—resonance mechanism. In contrast, their high-field (>1 *T*) polarization remains weak. In contrast, the major conformer signals (e.g., W4, W5, W6, F8) remain clearly polarized in both the *J*—resonance and Δ*g*—dominated regimes, contributing more uniformly across the field range.

**Figure 5:**
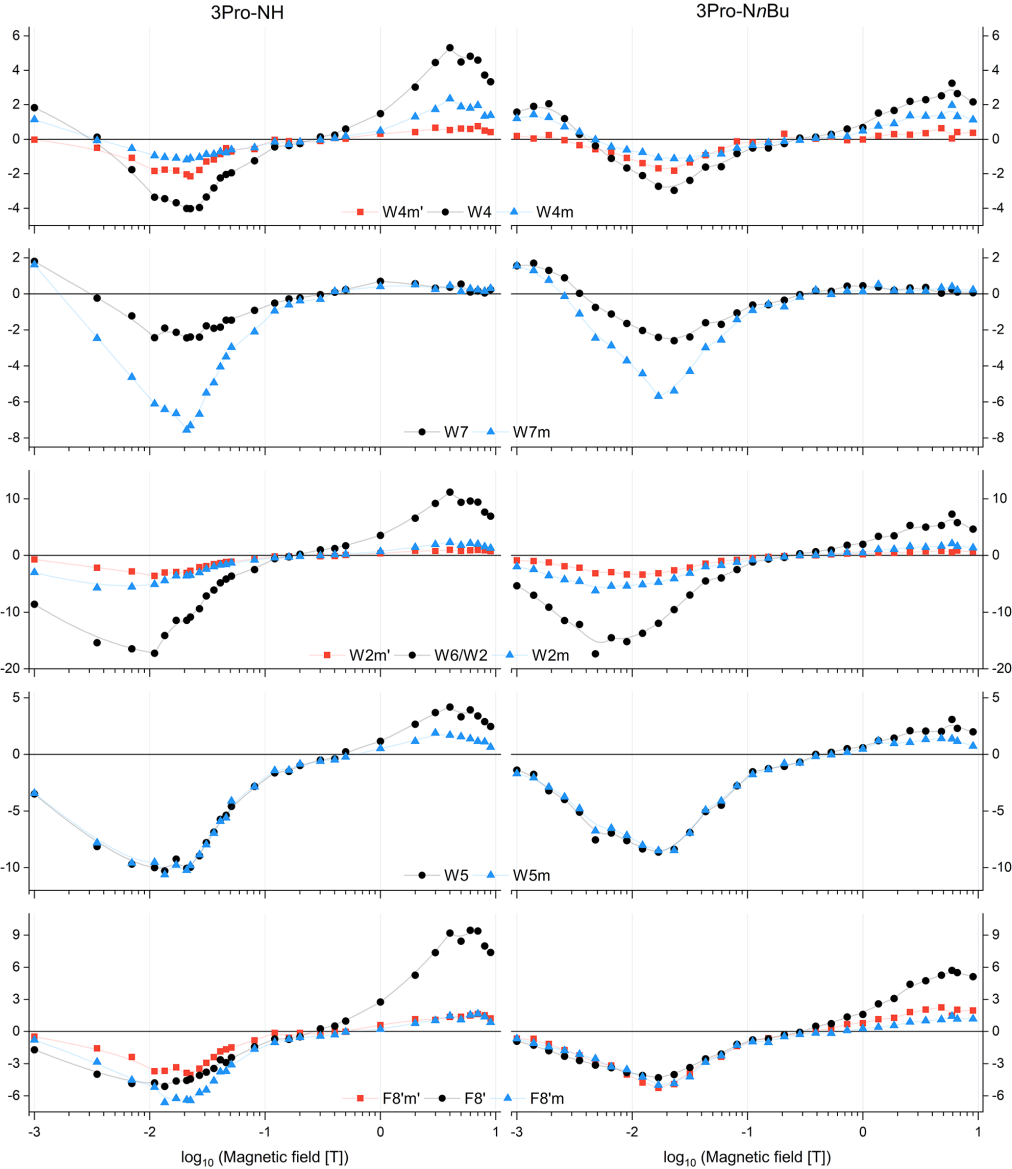
Magnetic field dependence of photo-CIDNP intensities for selected proton signals in the 3Pro-NH and 3Pro-N*n*Bu dyads 0.2 mM in MeOD-*d*_4_ at 293 K. Each plot shows the polarization amplitude of individual proton signals as a function of magnetic field, measured upon 450 nm LED irradiation. Signals from major conformers (e.g., F8′, W5, W6, W7) and minor conformers (labeled with “m”: e.g., W4_m_, W4_m′_, W7_m_, W6_m_) are tracked separately. Data are plotted versus log_10_ of the magnetic field (*T*); lines are used for guidance.

To further assess the role of intermolecular contributions, field-cycling photo-CIDNP experiments were performed at different dyad concentrations (see Figures S25–S27). Since LED irradiation enters the NMR sample from above, there is a trade-off between the spectral resolution, NMR signal sensitivity and the height of irradiated sample volume. The irradiation geometry is optimized for solutions with an optical density of approximately 1 cm^–1^, which corresponds to a dyad concentration of ∼0.1 mM for excitation at 450 nm. For samples with substantially higher optical density, strong CIDNP may still be generated within the irradiated region; however, this region may fall partially outside the sensitive volume of the NMR detection coil, leading to an apparent reduction of detected CIDNP intensity. In addition, photodegradation under continuous irradiation limits the useable concentration range. Therefore, most field-cycling experiments were carried out at ∼0.2 mM dyad concentration (optical density ≈2 cm^–1^), providing a compromise between efficient excitation and acceptable sample stability. Under these conditions, concentration-dependent field-cycling CIDNP measurements are feasible, but quantitative comparisons require careful normalization. In this case, the CIDNP field dependences at different dyad concentrations shown in Figures S25–S27 were normalized to the CIDNP intensity in the *J*—dominated low-field feature at about 10 mT.

An increase in concentration leads to a systematic enhancement of the Δ*g*—driven CIDNP contribution relative to the *J*—dominated low-field feature in 3Pro-N*n*Bu, 4Pro-NH dyads. This is consistent with the nature of the Δ*g* mechanism and can be explained by two main reasons: First, the Δ*g* contribution is more effective in the case of a bimolecular reaction, where radicals undergo diffusional separation. In contrast, in a monomolecular reaction, where radicals cannot diffuse apart, all nuclear-spin subensembles of triplet state gradually convert to singlet state, resulting in the cancellation of the steady-state. Second, the relative contribution of the Δ*g*-dominated regime increases with a higher initial concentration of geminate triplet radial pairs. These pairs are formed via the intermolecular electron transfer between the triplet excited dyad molecule F^T^-W and a diamagnetic dyad molecule F–W upon their encounter in the solution, with the rate of encounters being proportional to the dyad concentration. In contrast, no significant change in the shape of the CIDNP field dependence is observed for the 9Pro–N*n*Bu dyad upon increasing the concentration from 0.1 mM to 0.2 mM (Figure S27). These results demonstrate that the balance between intra- and intermolecular spin-selective pathways can be tuned by concentration and support the mechanistic assignments discussed above.

### Molecular Dynamics Simulations

The measured photo-CIDNP spectra point out that for most of the protons, peaks are present for the major and minor conformers of the studied dyads. The minor peaks for the F7′ and F8′ protons tend to disappear with the increasing proline chain length. A previous study^43^ utilized the 2D ^1^H–^1^H TOCSY and ROESY/NOESY spectra that indicated for the 4Pro-NH dyad the concentration of the PPII helix in MeOD-*d*_4_ was about 52%. For the dyads with longer proline chain, the concentration of the conformers with the PPII helix increases. The 2D spectra for the 3Pro-NH were unresolvable, which could indicate a substantial number of *cis*–isomers present in the solution. To investigate the possibility of minor conformers arising from the *cis* proline isomers, we conducted all-atom molecular dynamics (MD) simulations for the dyad 3Pro-NH. Eight possible structures with different combinations of *cis*– and *trans*–isomers in the proline chain were constructed and studied (e.g., *trans*–*trans*–*trans*, *cis*–*trans*–*trans*, etc.). The MD simulation procedure was consistent with our previous study,^43^ with the only difference being that the total number of molecules was kept constant across all 3Pro-NH simulations. The conformation of the prolines in the simulations was controlled with the peptide bond dihedral angles ω. The results of the MD simulations revealed no change between different conformations, except one (see details in Figure S26). For each simulation, the relative stability of different conformers was investigated, and the characteristic distances and orientations between W and F moieties were analyzed. Figure 6A shows the probability density distributions of energy, encompassing both the total energy of the dyads and the intermolecular interactions (Coulomb and van der Waals) with the solvent molecules, for the eight different characteristic configurations.

**Figure 6:**
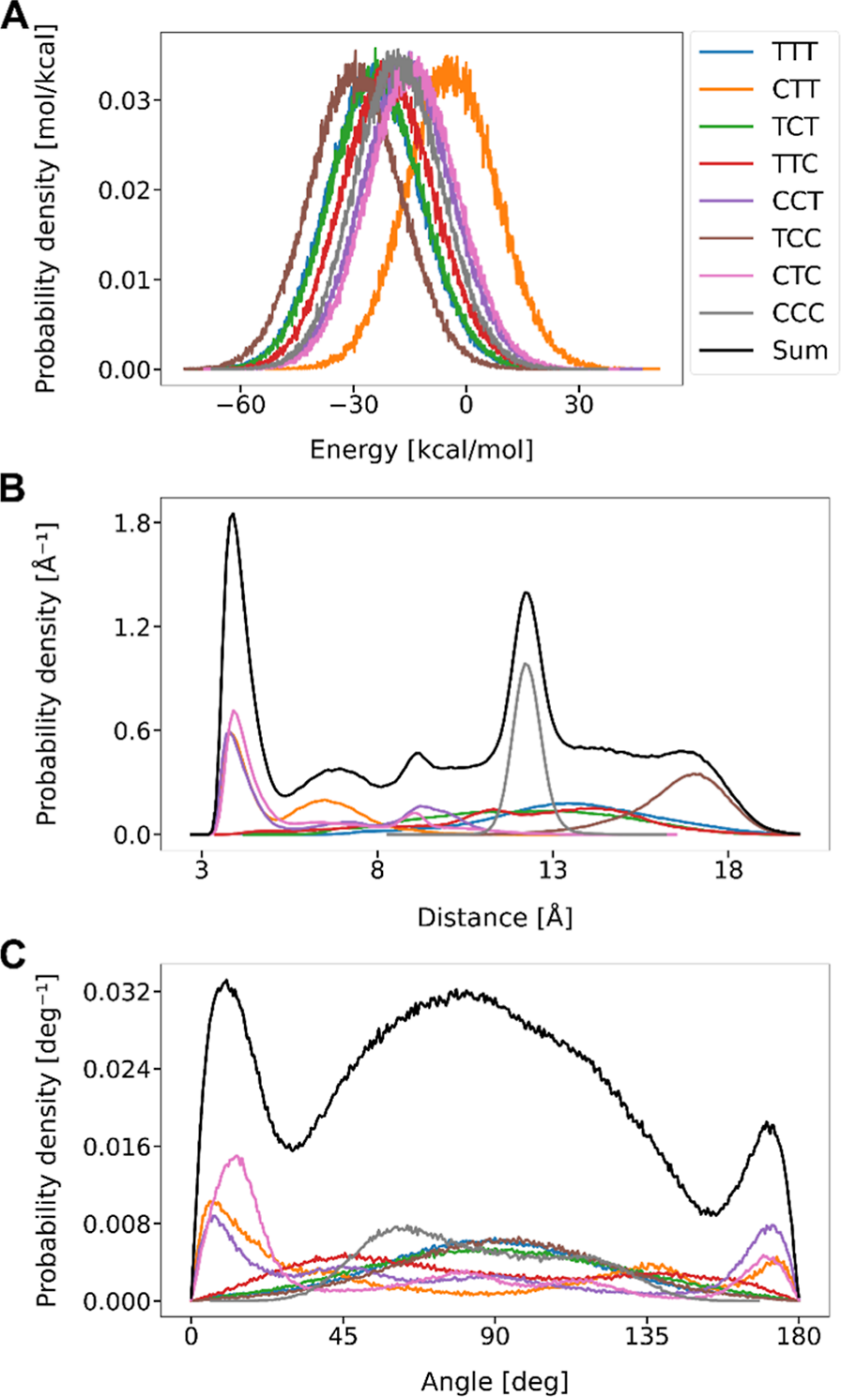
(A) Probability density distributions describing the possible 3Pro-NH energetics (see text for details) obtained from the MD simulations for eight different proline chain conformations. (B) Probability density obtained from MD simulations describing the distributions of possible distances between the geometric centers of F and W moieties in the 3Pro-NH dyads with the different proline conformations. (C) Probability density obtained from MD simulations describing the distributions of possible relative angles between the two normal vectors of the F and W moieties in the 3Pro-NH dyads with the different proline conformations. The letters “T” and “C” refer to *trans*– and *cis–*proline conformations, where numeration starts from the closest to the flavin. “Sum” illustrates the sum of the distributions for all configurations. Panel A does not contain the summed up probability distribution denoted as “Sum”.

The energy distributions for the different proline chain configurations peak around negative energy values, which indicates a favorable solvation of the dyads MeOD-*d*_4_. The Gaussian profiles of each conformer overlap closely with similar mean and variance values. The difference in the mean values between the most left and most right configurations (TCC and CTT) is approximately 26 kcal/mol for all 108 atoms for the 3Pro-NH dyad, which results in a difference of around 0.25 kcal/mol per atom. The similar energy values indicate that, in general, all eight conformers are probable and are likely to coexist in solution.

Similar to the previous study^43^ we have investigated the distance and relative orientations of the W and F moieties in the dyads to gain a deeper insight into the prominent structural motives that may explain the experimentally observed satellite peaks in the NMR spectrum for short dyads. Figure 6B,C show the probability distribution for the distance vector between the centers of the W and F and the angle between them for 3Pro-NH with each possible proline configuration. The angle is defined between the normal vectors to the F and W planes. Two prominent peaks are observed around 3–6.5 Å and 12 Å, where the latter is primary attributed to the CCC configuration. The former peak results due to multiple configurations and has the highest probability.

The conformations with distances between 3 and 6.5 Å and angles smaller than 45 and greater than 135° refer to the stacked structures, where F and W are close to each other and tend to the configuration with the angle of 0 or 180° (π–π stacking). Figure S28A shows the probabilities of F–W π–π stacking presence in the MD simulations for a specific proline conformation, reported as conditional probabilities for individual *cis* and *trans* proline configurations. The stacked motives are found in about 20% of cases relative to all possible proline conformations. The prominent presence of the stacked motives can be potentially connected to the minor satellite peak, for i.e., the F8′ signal that is significantly up-shielded in the photo-CIDNP spectra (see Figure S13A).

Another possible structural motive formed between F and W moieties that may lead to changes in chemical shifts is characterized by a hydrogen bond formation. Figure S28B shows the probability of the hydrogen bonds formed between the F oxygens and the W hydrogen atoms. The distance between the oxygen and hydrogen was chosen to be less than 3 Å, and the angles formed by N–H···O and the H···O–C atoms were considered to be greater than 130°.^63^ Hydrogen bonds between F and W altogether are formed in about 5% of cases relative to all possible proline conformations.

To analyze the chemical shielding behavior of the two selected structural motifs, such as hydrogen bonds and π–π stacking, an ensemble of the 25 energetically lowest-lying configurations was selected using CREST,^51^ following geometry optimization with TPSSh^64^/EPR-II.^65^ Figure 7A illustrates that most of the configurations are energetically close. The chemical shielding of the F7′ and F8′ hydrogens (as seen in Figure 7B) can be categorized into an up-shielding and down-shielding region based on their geometrical features. For instance, all studied conformers tend to inherit a strong up-shielding in comparison to a free flavin-linker system (see Figure 7C) for purely stacked configurations that lack a hydrogen bond between the F moiety and other functional groups. Conformers that exhibit a down-shielding character are also included in the chosen ensemble and additionally reveal a hydrogen bond with the NH_2_ group of the W, as illustrated in Figure 7C.

**Figure 7:**
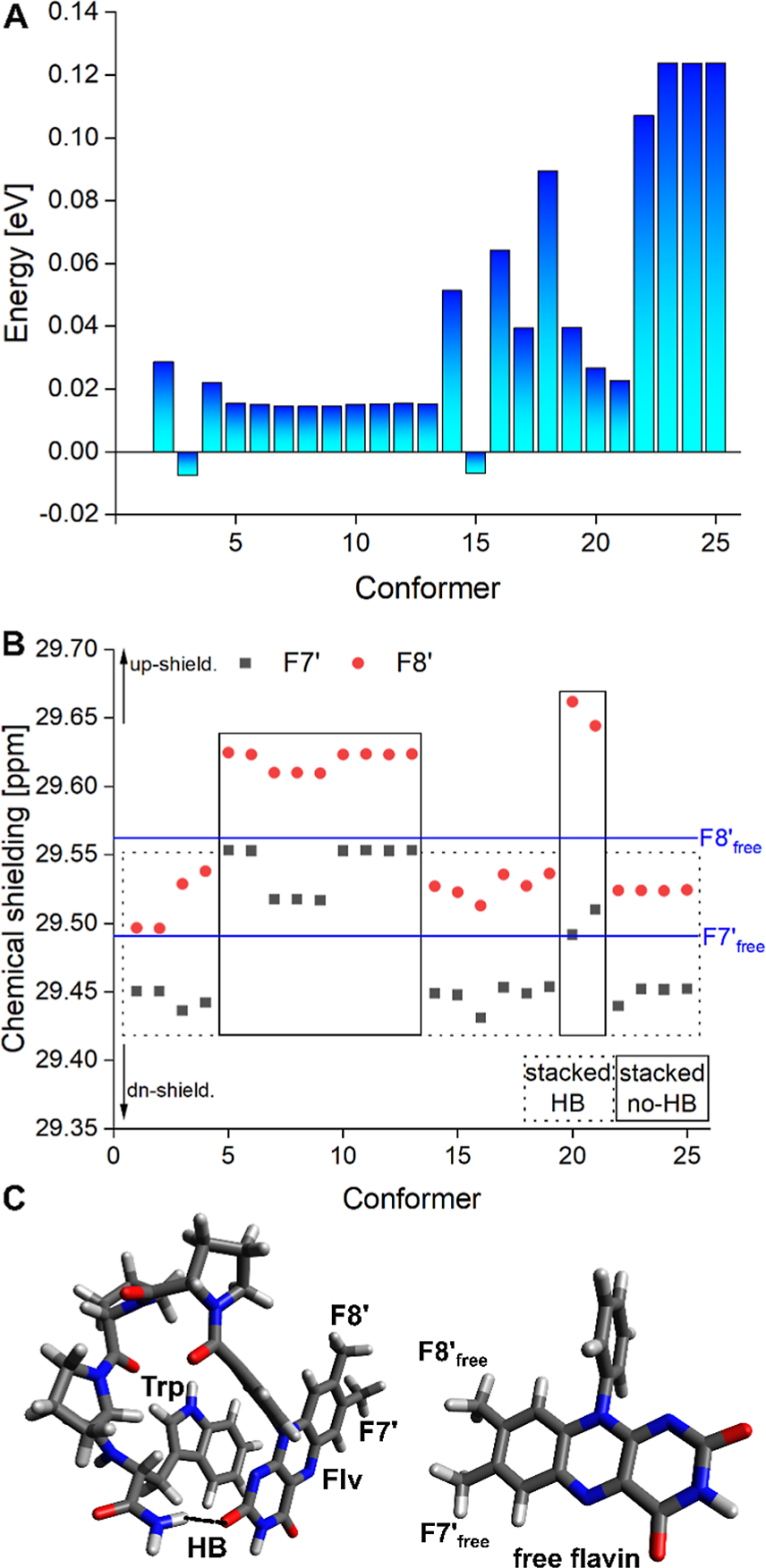
A) Relative energy of 25 selected 3Pro-NH dyad conformers. All conformers are energetically close. (B) Chemical shielding of F7′ and F8′ hydrogens for each conformer in comparison to a free flavin-linker system (blue lines). Strong up-shielding can be categorized as stacked conformers without hydrogen bonds, whereas down-shielding scenarios involve an additional hydrogen bond. (C) Illustration of stacked conformer with a hydrogen bond between the F moiety and the NH_2_ of the W. The free flavin-linker system, which is used as a reference, is illustrated additionally (right).

A further verification that π–π stacking causes up-shielding of the F7′ and F8′ hydrogens, while hydrogen bonds tend to promote down-shielding, is provided in the Supporting Information (see Figure S30). Here, a potential surface scan of various possible stacking configurations demonstrates a clear up-shielding in all scenarios. Thus, it can be concluded that the significant peaks at lower chemical shifts observed for F8′ are likely due to stacked moieties in the 3Pro-NH dyads, which are not found in longer dyads (see Figure 2) due to the larger distance between the F and W moieties.

## Discussion

Inspired by the active site of the LOV domain proteins, we have synthesized a series of F–W dyads connected by oligoproline linkers of varying length. Proline is unique among the natural amino acids due to its cyclic structure, in which the side chain forms a ring with the backbone nitrogen.^66^ This geometry enables *cis*–*trans* isomerization around the peptide bond preceding proline; although *cis*–*trans* isomerism can also occur in other peptides, the process is typically too fast to be detected on the NMR time scale. In contrast, the amide bond involving proline undergoes much slower isomerization due to its cyclic structure, making both rotamers observable in NMR spectra.^67^ Consequently, the *cis*–*trans* isomerization of the peptidyl–prolyl amide bond is a slow process on the NMR time scale and is associated with a relatively high activation barrier (∼20 kcal/mol).^46^ As a result, the proline residues in the oligoproline chains exist as a static mixture of conformers, each contributing independently to the observed CIDNP behavior.

Polyproline linkers can adopt two distinct helical conformations, depending on the *cis*–*trans* configuration of the peptidyl–prolyl amide bonds: the polyproline II (PPII) helix in the all-*trans* form, and the polyproline I (PPI) helix in all-*cis* form. In protic solvents such as water or methanol, they are generally expected to adopt the PPII conformation, with three prolines forming one helical turn and an approximate distance of ∼3.1 Å per residue, acting as rigid “molecular rulers”.^44,45^ Increasing the number of proline residues stabilizes the PPII helix, as observed by CD spectroscopy.^43^ In shorter linkers, however, helix formation is less favorable, and a higher fraction of prolines containing *cis*-amide bonds is present. This trend is clearly seen in the ^1^H NMR spectra (see Figure 2), where the peaks of W and F moieties become sharper and better resolved as the linker length increases.

Two-dimensional NMR further reveals that short dyads (e.g., 3Pro-N*n*Bu) display multiple conformers and minor signals, as well as the 6Pro-N*n*Bu dyad shows additional conformers that are overlapped in the 1D-^1^H spectra. For example, in the HSQC spectra, the F6, W5, and W2 positions exhibit three or more distinct cross-peaks, indicating multiple slowly exchanging conformations (see Figures S7–S9).

The coexistence of both “*cis*” and “*trans*” proline conformers introduces structural diversity, potentially influencing the electronic coupling and spin dynamics of the dyads.^68^ It also complicates the analysis of 1D spectra since each observed peak may consist of contributions from several unresolved conformations. Notably, such conformational heterogeneity provides a natural explanation for the multiple CIDNP pathways observed at different fields: *cis*–rich compact conformers favor short-range encounters and biradical recombination, while extended *trans* conformers contribute predominantly in the Δ*g*—dominated regime.

A detailed analysis of the conformational states was presented in our previous work using 2D-^1^H–^1^H TOCSY and ROESY/NOESY spectra across the whole series of dyads.^43^ In each system, we highlighted the percentage of PPII helices. For example, the 4Pro-NH and 6Pro-NH dyads exhibited distinct species, with population distributions of approximately 52% PPII (all-*trans*) and 48% *cis*–containing isomers, and 61% PPII and 39% *cis*–containing isomers, respectively. Moreover, the comparison between NH- and N*n*Bu -substituted dyads confirmed that alkylation did not significantly affect the conformational preferences. Even the 9Pro-NH dyad retained ∼30% minor isomer content. These results explain the observed spectral complexity in dyads and support a model in which proline isomerization modulates the local environment of the F and W labels.

Previous studies have shown that the presence of a *cis*–proline “defect” within an oligoproline chain reduces the donor–acceptor distance, resulting in a more compact molecular geometry.^44,68,69^ In particular, when the *cis* residue is located in the middle of the chain, the donor and acceptor are brought significantly closer together.^68^ Our DOSY results for the 3Pro-N*n*Bu dyad are consistent with this finding: the “m” conformer exhibits a higher diffusion coefficient and a shorter rotational correlation time than the major conformer, indicating a more compact structure in solution (Figure S13). This supports the assignment of the “m” conformer to a *cis*–containing-type geometry, which would shorten the interchromophore distance relative to the all-*trans* PPII form. Notably, no aggregation effects are apparent for 3Pro-N*n*Bu, likely because the short linker allows intramolecular folding in which one chromophore (flavin) interacts with the tryptophan moiety of the same molecule, as also supported by our MD simulations.

In contrast, the lower diffusion coefficient and longer correlation time observed for the minor conformer F8′_m_ in the 9Pro-N*n*Bu dyad may originate from intermolecular aggregation. Aggregation of flavins in solution has been previously detected by diffusion-ordered spectroscopy (DOSY), where the measured diffusion coefficient reflected the number of aggregated molecules.^70^ Furthermore, ^1^H NMR chemical shift analysis demonstrated that self-association of flavin mononucleotide (FMN^2–^) leads to systematic shifts of flavin proton resonances,^71^ which could explain the signal displacement observed here. In addition, our MD simulations revealed that different stacking arrangements (flavin–flavin and flavin–tryptophan) can cause significant changes in the NMR chemical shifts of the involved protons, supporting the hypothesis that aggregation effects contribute to the observed NMR signatures of the minor conformer.

The time-resolved CIDNP kinetics indicate that, except for the 4Pro-NH dyads, all systems exhibit a significant intermolecular contribution to the polarization formation. For these dyads, the kinetic profiles cannot be explained solely by intramolecular radical pair recombination; instead, the data are consistent with a mechanism involving encounters between flavin- and tryptophan-centered radicals from different molecules. This intermolecular pathway becomes more prominent with increasing linker length, as the probability of intramolecular electron transfer decreases and intermolecular encounters dominate the polarization dynamics. These kinetic results are in complete agreement with the field-dependent CIDNP measurements: the absence of *J*—resonance CIDNP in the dyads with 6–12 proline and the similarity of their profiles to the unconjugated reference confirm that intermolecular pathways dominate when the linker is long.

Having established the structural origin of minor and major conformers, we now turn to the magnetic field dependence, which provides the most direct mechanistic fingerprint of biradical versus Δ*g*—driven pathways.

Magnetic field dependence of CIDNP is a highly sensitive tool for detecting biradicals, since strong polarization is formed at the level anticrossing (LAC), following the condition

5 $$B_{LAC}\approx \frac{\left|J_{ex}\right|}{g\mu_{B}}$$

which results from the hyperfine-induced singlet–triplet mixing. The characteristic features of CIDNP generated via biradical recombination are (i) the same polarization sign for all observed nuclei, (ii) a pronounced maximum at *B*_LAC_ (often, though not necessarily, exceeding typical hyperfine fields), and (iii) weak or absent response in the high-field Δ*g*—dominated regime, where scavenging processes still occur but their contribution to spin-selective biradical recombination (and thus to CIDNP) becomes negligible.

All three criteria are fulfilled in our experiments for the minor conformers of the 3Pro-NH/N*n*Bu and 4Pro-NH dyads. In these cases, strong CIDNP effects were observed at *B*_LAC_ = 5–20 mT, consistent with recombination of geminate biradicals. The uniform emissive polarization across all monitored protons confirms the biradical origin of these signals. Hence, the same minor-conformer resonances are significantly weaker at high magnetic fields (≥1 T, see Figure 5), which further supports the assignment: Δ*g*—driven polarization dominates in that regime, but biradical recombination contributes negligibly.

An additional complication arises because chemical shifts of *cis*–and *trans*–containing conformers often overlap. As a result, signals attributed to major *trans* conformers in the high-field Δ*g*—dominated regime may in fact contain contributions from *cis*–rich compact conformers, whose NMR signal positions coincide with those of the dominant *trans* population. This explains why some “major” signals appear to display both high-field Δ*g*—type CIDNP and, at the same time, low-field *J*—resonance behavior.

## Conclusion

*Cis*–proline–containing compact structures, which correspond to minor conformers, constitute the principal source of *J*—resonance CIDNP in these systems. Such geometries favor short donor–acceptor distances, enhance the exchange interaction, and shift the level anticrossing condition into the experimentally observed 5–20 mT region. By contrast, dominant *trans*–rich major conformers contribute primarily at high magnetic fields through Δ*g*—bimolecular mechanisms, and do not exhibit pronounced low-field *J*—resonance enhancement.

Field-cycling photo-CIDNP therefore proves to be exquisitely sensitive to conformational heterogeneity and can selectively detect minor, transient molecular states that are often inaccessible to conventional structural methods. In the flavin-tryptophan/polyproline model system, rare *cis*-containing linker conformers generate compact donor–acceptor geometries with enhanced electronic exchange, enabling intramolecular biradical recombination and the emergence of characteristic *J*-resonance CIDNP signatures at low magnetic fields. In contrast, extended all-*trans* (PPII) conformers predominantly give rise to intermolecular radical encounters, leading to Δ*g*-dominated polarization behavior at high field. Proline *cis*–*trans* isomerization thus functions as a structural switch that governs not only donor–acceptor distance, but also the balance between fundamentally different spin-selective reaction pathways.

Beyond the specific flavin-tryptophan/polyproline model system, our findings establish a general principle: small populations of compact conformers can dominate spin-dependent reactivity and polarization, despite representing only a minor fraction of the ensemble. This insight has important implications for flexible redox-active molecules and biomolecular assemblies, where conformational fluctuations, transient contacts, and weak intramolecular interactions are ubiquitous.^72^ Field-cycling photo-CIDNP therefore emerges as a powerful mechanistic probe that complements traditional structural techniques by reporting directly on the spin dynamics of short-lived biradical states and their structural origins.

More broadly, the results strengthen the conceptual foundation of photo-CIDNP in covalently linked donor–acceptor systems by unambiguously linking magnetic-field-dependent polarization patterns to specific conformational subensembles. By demonstrating how conformational dynamics can gate spin chemistry, our study contributes to a deeper understanding of structure-dynamics-function relationships in photochemical and redox processes, with potential relevance ranging from the design of molecular spintronic elements to the interpretation of spin effects in biological electron-transfer systems.

## Supplementary Material



## Notes

Additional information on Materials and Methods, including details on sample preparation; UV–Vis absorption protocols; standard NMR measurement protocol; light-driven NMR (time-resolved photo-CIDNP) and field-cycling measurement details and pulse sequences; molecular dynamics simulation protocols; additional spectral information: ^1^H chemical shift assignment, ^1^H–^13^C HSQC spectra in MeOD-*d*_4_ for 3Pro-N*n*Bu and 6Pro-N*n*Bu; diffusion ordered spectroscopy data, relaxation-dispersion data sets, signal-processing notes, rotational correlation time table, time-resolved photo-CIDNP ^1^H spectra, kinetic data from time-resolved photo CIDNP experiments, and field-cycling photo-CIDNP profiles for N*n*Bu dyads, ^1^H photo-CIDNP NMR spectra of all dyads at high field, field-cycling photo-CIDNP profiles comparison for NH and N*n*Bu dyads, ^1^H photo-CIDNP NMR spectra at different magnetic field for 3Pro-NH and -N*n*Bu dyads, field profiles of W8 protons of tryptophan, as well as extended MD results. (PDF)

## Notes

This article is dedicated to the late Prof. Hans-Martin Vieth.
