# Supplementary material for: Conformational Switching Controls Biradical Spin Dynamics in Flavin–Tryptophan Dyads
Source: J Am Chem Soc. 2026 Mar 23;148(15):15897–910. doi: 10.1021/jacs.5c22947 (PMC13107450; doi:10.1021/jacs.5c22947)
Supplement: Supplementary file 1 [file ja5c22947_si_001.pdf]

Conformational Switching Controls Biradical Spin Dynamics in Flavin–Tryptophan Dyads
(Supporting Information)

Guzel Musabirova[1*], Olga B. Morozova[2], Alexey S. Kiryutin[2], Irina S. Anisimova[3], Ivan V. Zhukov[2], Tobias Theiss[4], Luca Gerhards[3], Ben G. E. Zoller[5], Tanja Gulder[4,5,7], Jörg Matysik[1], Ilia A. Solov'yov[3,6,8], Alexandra V. Yurkovskaya[2*]

[1] Department of Analytical Chemistry, Leipzig University, Linnéstr. 3, 04103, Leipzig, Germany

[2] International Tomography Center, Siberian Branch of the Russian Academy of Science, Institutskaya 3A, 630090, Novosibirsk, Russia

[3] Institute of Physics, Carl von Ossietzky Universität, Carl-von-Ossietzky-Str. 9–11, 26129, Oldenburg, Germany

[4] Department of Organic Chemistry, Leipzig University, Johannisallee 29, 04103, Leipzig, Germany

[5] Organic Chemistry, Saarland University, 66123, Saarbrücken, Germany

[6] Research Center Neurosensory Science, Carl von Ossietzky-Universität, Carl-von-Ossietzky-Str. 9–11, 26129, Oldenburg, Germany

[7] Synthesis of Natural-Product Derived Drugs, Helmholtz Institute for Pharmaceutical Research Saarland (HIPS) Helmholtz Centre for Infection Research (HZI), 66123, Saarbrücken, Germany

[8] Center for Nanoscale Dynamics (CENAD), Carl von Ossietzky-Universität, Carl-von-Ossietzky-Str. 9–11, 26129, Oldenburg, Germany

* Guzel Musabirova

Email: guzel.musabirova94@gmail.com

* Alexandra V. Yurkovskaya

Email: yurk@tomo.nsc.ru

Contents

1. Materials and methods 3
   1.1. Sample preparation 3
   1.2. UV-Vis Absorption Spectroscopy 4
   1.3. Standard NMR Measurements 4
   1.4. NMR experiments using light 5
      1.4.1 Time-Resolved photo-CIDNP measurements 5
      1.4.2 Field-Cycling measurements 6
   1.5. Molecular dynamics simulations protocol 8
2. Additional spectral information 9
3. Diffusion ordered spectrosopy 12
4. Relaxation dispersion 13
   4.1 Signal processing 14
5. Time-Resolved photo-CIDNP 20
6. Field-cycling photo-CIDNP 23
7. Additional results from molecular dynamics simulations 34
8. References 37

1. Materials and methods

1.1. Sample preparation

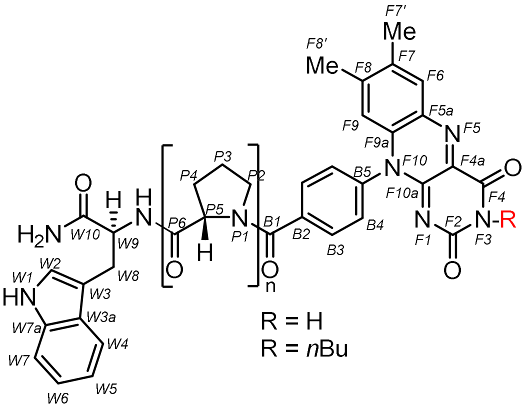

Figure S1. General structure of the studied Trp–Pro_n–F dyads. The dyads consist of a tryptophan electron donor (W), a polyproline spacer (P, n = 3–12), and a flavin electron acceptor unit (F). Two series were studied: R = H with proline units n = 3, 4, 6, 9, 12, and R = *n*Bu with proline units n = 3, 6, 9, resulting in eight dyads in total.

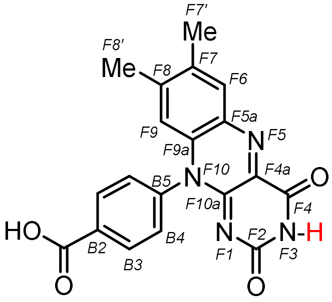

Figure S2. General structure of the N10-(4-carboxyphenyl)-flavin investigated as a reference system in this work.

The studied W–Pro_n–F dyads were synthesized as previously reported[1]. The dyads were dissolved in MeOH-*d₄* (99.98% purity), which was purchased from Carl Roth GmbH & Co. KG, Karlsruhe, Germany, for UV-VIS and NMR spectroscopy. For standard NMR measurements, dyads were dissolved in MeOD-*d₄* at a high concentration of approximately 10 mM. For a photo-CIDNP field- and time-dependent measurements, all dyads were dissolved in a concentration to achieve an optical density of ~2 at 450 nm per cm, which corresponds to ~0.2 mM. Before measurements, the samples were purged with argon for 6-10 minutes to remove dissolved oxygen. Only data acquired on freshly prepared, minimally photobleached samples were used for the final analysis. Measurements on pre-irradiated samples were excluded because of a significant loss of signal intensity.

1.2. UV-Vis Absorption Spectroscopy

UV-Vis absorption spectra were recorded on an Agilent 8453 UV-visible spectroscopy system. Measurements were carried out in quartz cuvettes with a path length of 0.5 cm.

Samples were prepared by dissolving the compounds in MeOH-d_4 at concentrations of ~0.2 mM unless stated otherwise. Spectra were collected in the range of 200–600 nm with a spectral resolution of 1 nm. Baseline correction was performed using the corresponding solvent as a reference.

1.3. Standard NMR Measurements

For all NMR measurements, chemical shifts were referenced to the residual solvent signals (δ_H = 3.31 ppm, δ_C = 49.0 ppm for MeOD-d_4).

For the ^{1}H 1D NMR experiments, a Bruker-Spectrospin 600 UltraShield NMR spectrometer (Bruker Corporation, Karlsruhe, Germany) was used with a 5 mm TBI probe (PH TBI600S3 H/C-BB-D-05 Z LTB). Experiments were performed at 293 K. For the acquisition of thermally polarized ^{1}H spectra, a standard pulse sequence was employed.

All the following experiments were performed on a Bruker Avance III HD 700 MHz spectrometer equipped with a TXI probe and a custom-built fast field-cycling (FFC) shuttle system, similar to that described previously [2].

Two-dimensional ^{1}H–^{13}C HSQC spectra were recorded using the standard gradient-selected pulse sequence (hsqcetgp). Typical acquisition parameters were: number of complex points = 4096 (^{1}H) × 512 (^{13}C), relaxation delay = 3 s, and 32 scans per increment.

Diffusion measurements were performed using the stimulated echo (STE, Bruker pulse program *stegp1s*). The gradient strength was incremented linearly in 16–32 steps from 2% to 95% of the maximum gradient amplitude (up to 50 G/cm). The diffusion delay (Δ) was set to 50-100 ms, and the gradient pulse duration (δ) was 2-4 ms. The longitudinal relaxation delay was 2 s, and 64 scans were collected per gradient step. DOSY spectra were processed in Bruker TopSpin 4.0 using the DOSY module based on the Stejskal–Tanner equation. Diffusion coefficients (D) were extracted by fitting the decay of signal intensities as a function of gradient strength using monoexponential fitting.

Measurements of the relaxation time T_1 were performed using the inversion recovery method on a custom-built setup designed for rapid vertical displacement of the sample within the cryomagnet [2]. The construction of the 700 MHz shuttle closely follows the design of our earlier setup for the 400 MHz spectrometer [2]; technical details specific to the 700 MHz version will be presented elsewhere.

The magnetic field for relaxation was controlled by the sample's position relative to the center of the cryomagnet bore. The experimental procedure was as follows (see Figure S3):

1. The sample was relaxed in a 16.4 T magnetic field for 15 seconds to establish the initial Boltzmann polarization.

2. A 180-degree hard pulse was applied to invert the magnetization of all protons.

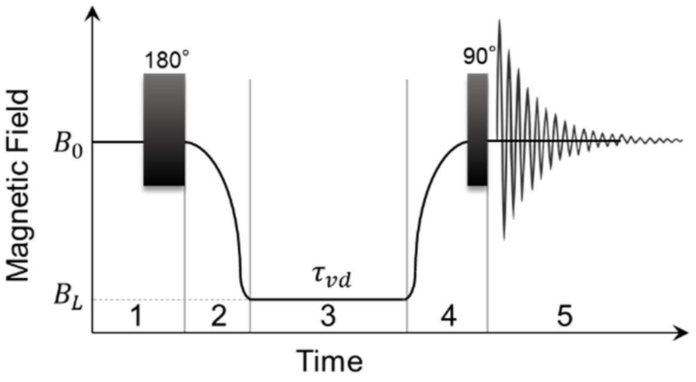

Figure S3. Experimental protocol for measuring nuclear spin relaxation dispersion. In step 1, spins relax to thermal equilibrium at high field $\boldsymbol{B_0}$, then a 180° RF pulse is applied for inversion of magnetization. In step 2, the sample is transferred to the field $\boldsymbol{B_L}$, where it is kept for an incremented delay $\boldsymbol{\tau_{vd}}$ during step 3. In step 4, the sample is transferred back to high field $\boldsymbol{B_0}$ and a 90° RF pulse is applied. In step 5, the nuclear spin free induction decay is acquired, and the NMR spectrum is obtained after FT.

3. The sample was rapidly moved (within 300 ms) to the desired magnetic field position for relaxation.

4. The sample was held in the relaxation field (B_0) for a variable time interval ranging from 0.05 to 10 seconds. (16-time delays: 0.05; 0.071; 0.101; 0.144; 0.205; 0.292; 0.416; 0.593; 0.844; 1.201; 1.71; 2.434; 3.466; 4.934; 7.024; 10)

5. After relaxation, the sample was moved back to the detection field.

6. A delay of 200 ms was included to allow vibrations of the sample to subside.

7. Finally, the NMR signal was recorded using a 90-degree pulse followed by free induction decay (FID) detection.

8. To achieve an acceptable signal-to-noise ratio, 4 scans were averaged for each measurement.

9. Relaxation measurements were performed in magnetic fields ranging from 0.1 T to 16.4 T, with the number of field values varying between 30 and 50.

1.4. NMR experiments using light

1.4.1 Time-Resolved photo-CIDNP measurements

The instrumentation for TR-CIDNP experiments has been described previously[3]. Briefly, samples were degassed by bubbling with pure nitrogen in standard Pyrex NMR ampoules. Measurements were carried out on a Bruker DPX-200 spectrometer operating at 200 MHz for protons (magnetic field 4.7 T). Excitation was provided by the third harmonic of a Quantel Brilliant B Nd:YAG laser (λ = 308 nm, pulse duration ≈ 20 ns, pulse energy 70–80 mJ). The

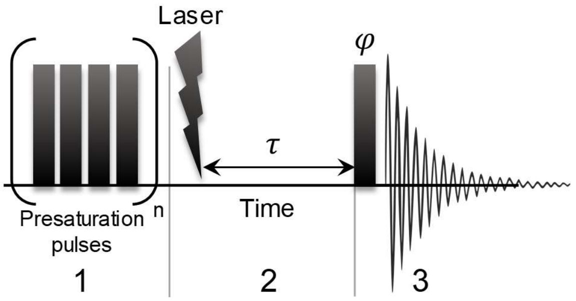

Figure S4. Experimental protocol for measuring the time-resolved photo-CIDNP. In stage 1, saturation with broadband radio frequency pulses; at step 2, the 20 ns laser flash with following variable delay τ is applied; at step 3, the detecting RF pulse (2 or 4 μs duration) is applied, and nuclear spin free induction decay is acquired. Duration of 90° RF pulse in this setup is 6 μs. To obtain the time-resolved photo-CIDNP kinetics that are shown in Fig. S15 we used 30° RF pulse (2 μs), and 60° RF pulse (4 μs) for kinetics shown in Fig. 3 of the main text.

laser beam was directed to the NMR sample through a prism-based optical arrangement and a 5 mm diameter quartz light guide.

TR-CIDNP spectra were recorded using the following pulse sequence (see Figure S4): (1) saturation of proton spins by broadband RF pulses, (2) application of a ~20 ns laser pulse synchronized with the NMR spectrometer trigger, and (3) detection with a 2 or 4 μs RF pulse followed by FID acquisition. The laser pulse was timed with the leading edge of the RF sequence. Since Boltzmann magnetization was effectively suppressed by the initial saturation, only signals arising from spin polarization generated in the photochemical cycle were observed in the TR-CIDNP spectra.

1.4.2 Field-Cycling measurements

Magnetic field–dependent photo-CIDNP measurements were performed on a Bruker Avance III HD 400 MHz spectrometer equipped with a field-cycling accessory and a custom LED irradiation module [2,4]. Illumination of the sample was achieved through a 4 mm quartz light guide positioned inside the NMR tube with its polished end located just above the RF coil. At the opposite end, a 450 nm LED (20 mW, heat-sink cooled) was coupled to the light guide. The LED operation was controlled by TTL pulses from the NMR console, ensuring synchronization with the RF sequence and sample shuttling.

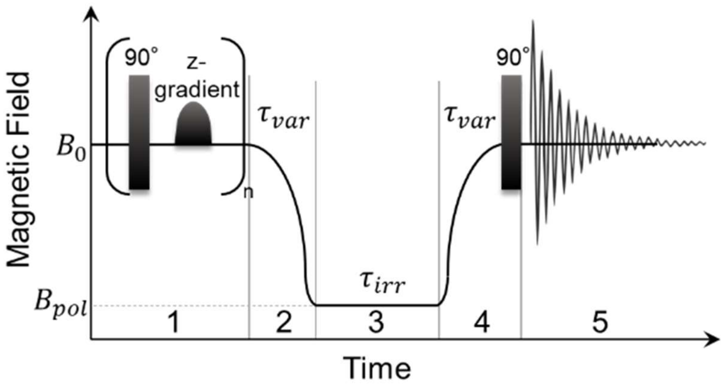

Figure S5. Experimental protocol for measuring the CIDNP field dependence. In stage 1 suppression of the thermal magnetization at magnetic field $\boldsymbol{B_0}$ 9.4 T. Then, in stage 2, mechanical transfer of the NMR sample to a variable magnetic field $\boldsymbol{B_{pol}}$ ($\boldsymbol{\tau_{var}} \leq 0.5$ s). Then at step 3, the LED is either switched on (in "LED ON" experiment) or stayed switched off (in "LED OFF" experiment) for $\boldsymbol{\tau_{irr}}$= 1 s. In stage 4, the sample is transferred to high field $\boldsymbol{B_0}$ and 90° RF pulse is applied. In stage 5, nuclear spin-free induction decay is acquired.

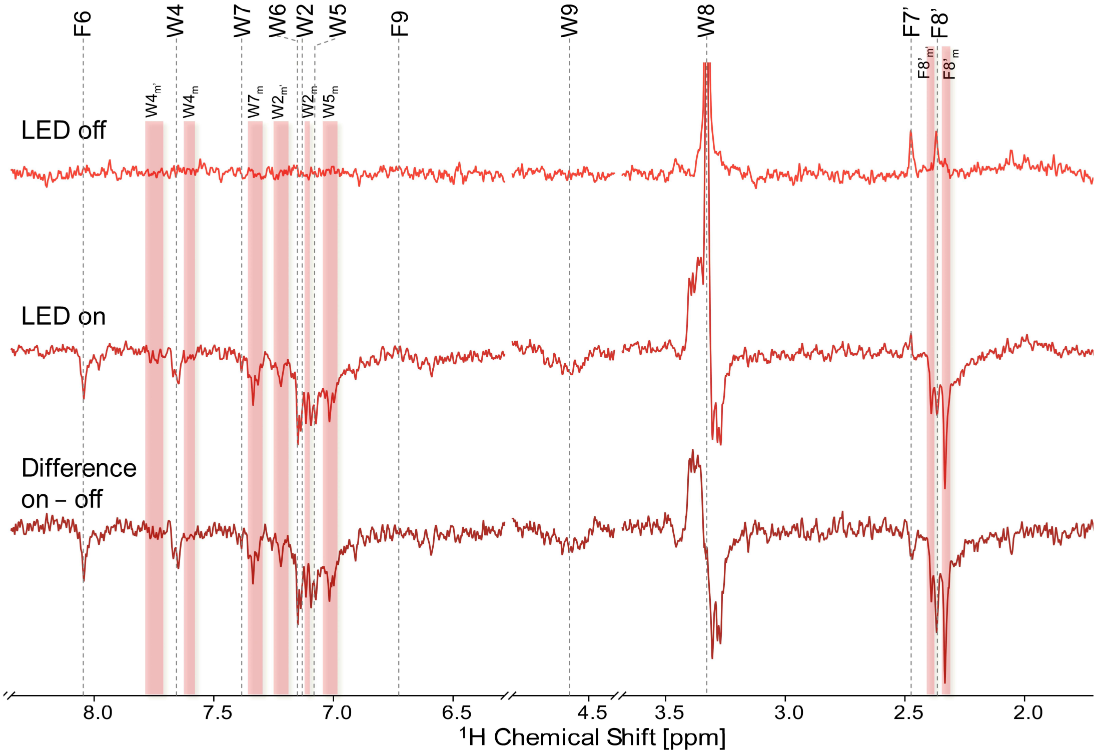

Figure S6. Dark, light, and difference ^{1}H NMR spectra of dyad 3Pro-NH in MeOD-d$_4$ at 293 K. The top spectrum was recorded in the dark, the middle at 17 mT under 1 s irradiation with 450 nm (20 mW) LED light, and the bottom trace shows the resulting light − dark difference spectrum.

The experimental sequence (see Figure S5) consisted of the following steps: (1) suppression of residual magnetization using a series of 90° and z-gradient pulses, (2) transfer of the sample to the selected low field B_{pol}, where (3) illumination with the LED was applied for τ_{irr} = 1 s, (4) return of the sample to the high field B_0 and (5) acquisition of the FID following a hard 90° pulse. To remove signals from Boltzmann magnetization, pairs of spectra were acquired with the LED on and off, and their difference yielded the photo-CIDNP spectrum. Field profiles of the photo-CIDNP effect were obtained at 30 logarithmically spaced field values between 1 mT and 10 T. A representative spectrum of the 3Pro–NH conjugate recorded at B_{pol} = 17 mT is shown in Figure S6.

All NMR data were analyzed using Bruker's TopSpin 4.0.9, MestReNova 14.2.1, and Origin 9.6.0.172 software.

1.5. Molecular dynamics simulations protocol

Molecular dynamics (MD) simulations of the 3Pro-NH dyads were conducted in accordance with the previous study[1] using NAMD [5,6]. Eight possible proline conformation of 3Pro-NH dyad were constructed (*trans-trans-trans, cis-trans-trans*, etc). As initial dyad configurations in the MD simulations, six different conformers were found for each proline conformation using CREST (Conformer–Rotamer Ensemble Sampling Tool) [7]. All the resulting structures were solvated in a rectangular simulation box of CD_3OD. All the parameters of the MD simulations were kept consistent with those used previously [1]. The only methodological difference between the current MD simulations and from the earlier study [1] was that the total number of molecules was kept constant across all of the 3Pro-NH solvated structures. The equilibration and simulation procedures were performed similarly to those reported in ref[1], resulting in a total production simulation time of 1.44 µs for each proline conformation.

Specific conformers of the 3Pro-NH dyads for the evaluation of chemical shielding were received using the CREST) algorithm [7]. Here, a temperature of 400 K was chosen for a better sampling of the phase space. Additionally, an implicit solvent of methanol was employed. The 25 energetically most stable structures in an energy window of 15 kcal/mol were extracted and post-optimized using the hybrid functional TPSSh and the EPR-II basis set implemented in ORCA 6.0 [8]. For the post-optimization procedure, an implicit solvent model of methanol within the CPCM framework was used.

2. Additional spectral information

Table S1. ^{1}H NMR chemical shifts δ for all dyads and N10-(4-carboxyphenyl)-flavin and tryptophan mixture in MeOD-d_4.

^{1}H Chemical shift [ppm]									
Protons	3Pro-NH	4Pro-NH	6Pro-NH	9Pro-NH	12Pro-NH	3Pro-N*n*Bu	6Pro-N*n*Bu	9Pro-N*n*Bu	Flv_Trp
F6	8.05	8.05	8.05	8.05	8.05	8.06	8.06	8.06	8.06
$F6_m$	8.04	8.03	8.03	8.03	8.03	8.05	8.04	8.04	—
B3	7.94,<br>7.92	7.95,<br>7.94,<br>7.93	7.96,<br>7.95	7.96,<br>7.95	7.96,<br>7.95	7.94,<br>7.92	7.96,<br>7.95,	7.96,<br>7.95,	8.35
B3′	7.91,<br>7.89	7.92,<br>7.91,<br>7.90	7.92,<br>7.91	7.92,<br>7.91	7.92,<br>7.91	7.91,<br>7.89	7.92,<br>7.91	7.92,<br>7.91	8.33
$W4_{m'}$	7.76,<br>7.75	7.69,<br>7.68	7.68,<br>7.66	7.67,<br>7.66	7.67,<br>7.66	7.76	7.67,<br>7.66	7.67,<br>7.65	—
W4	7.66.<br>7.65	7.65,<br>7.64	7.65,<br>7.63	7.64,<br>7.63	7.64,<br>7,63	7.66,<br>7.65	7.65,<br>7.63	7.64,<br>7.63	7.16,<br>7.14,<br>7.12
$W4_m$	7.63,<br>7.62	7.63,<br>7.62	—	—	—	7.63,<br>7.62	—	—	—
B4	7.56,<br>7.54	7.56,<br>7.55	7.57,<br>7.55	7.57,<br>7.56	7.57,<br>7.56	7.55,<br>7.54	7.56,<br>7.55	7.56,<br>7.55	7.39,<br>7.37
W7	7.40,<br>7.39	7.39,<br>7.38	7.39,<br>7.38,<br>7.36	7.39,<br>7.37,<br>7.35	7.39,<br>7.37,<br>7.35	7.40,<br>7.39	7.39,<br>7.37	7.38,<br>7.37	7.09,<br>7.07,<br>7.05
$W7_m$	7.36,<br>7.34,<br>7.33	—	—	—	—	7.35,<br>7.34,<br>7.33	—	—	—
$W2_{m'}$	7.22	—	—	—	—	7.22	—	—	—
W6/W2	7.17,<br>7.16,<br>7.15,<br>7.14	7.16,<br>7.15,<br>7.14	7.16,<br>7.15,<br>7.13,<br>7.12	7.15,<br>7.14,<br>7.13	7.15,<br>7.14,<br>7.13	7.17,<br>7.15,<br>7.14	7.14,<br>7.13,<br>7.12	7.15,<br>7.14,<br>7.13	7.73,<br>7.71,<br>7.21
$W2_m$	7.12	7.11	—	—	—	7.12	—	—	—
W5	7.10,<br>7.09,<br>7.08,<br>7.06	7.08,<br>7.07,<br>7.06,<br>7.04	7.08,<br>7.06,<br>7.05,<br>7.04,<br>7.03	7.07,<br>7.06,<br>7.05	7.07,<br>7.06,<br>7.05	7.09,<br>7.08,<br>7.07	7.07,<br>7.06,<br>7.05,<br>7.04	7.07,<br>7.06,<br>7.05	7.55,<br>7.53
$W5_m$	7.02	7.02	—	—	—	7.03,<br>7.02	—	—	—
F9	6.72	6.73	6.73	6.73	6.73	6.73	6.74	6.74	6.74
F7′	2.48	2.48	2.48	2.48	2.48	2.48	2.48	2.48	2.48
$F8'_m$	2.39	2.38	2.39	—	—	2.39	2.38	2.38	—
F8′	2.37	2.37	2.37	2.37	2.37	2.37	2.37	2.37	2.38
$F8'_{m'}$	2.34	2.36	2.36	2.36	—	2.34	2.36	2.36	—

^{1}H-^{13}C HSQC spectra of dyads in MeOD-d_4

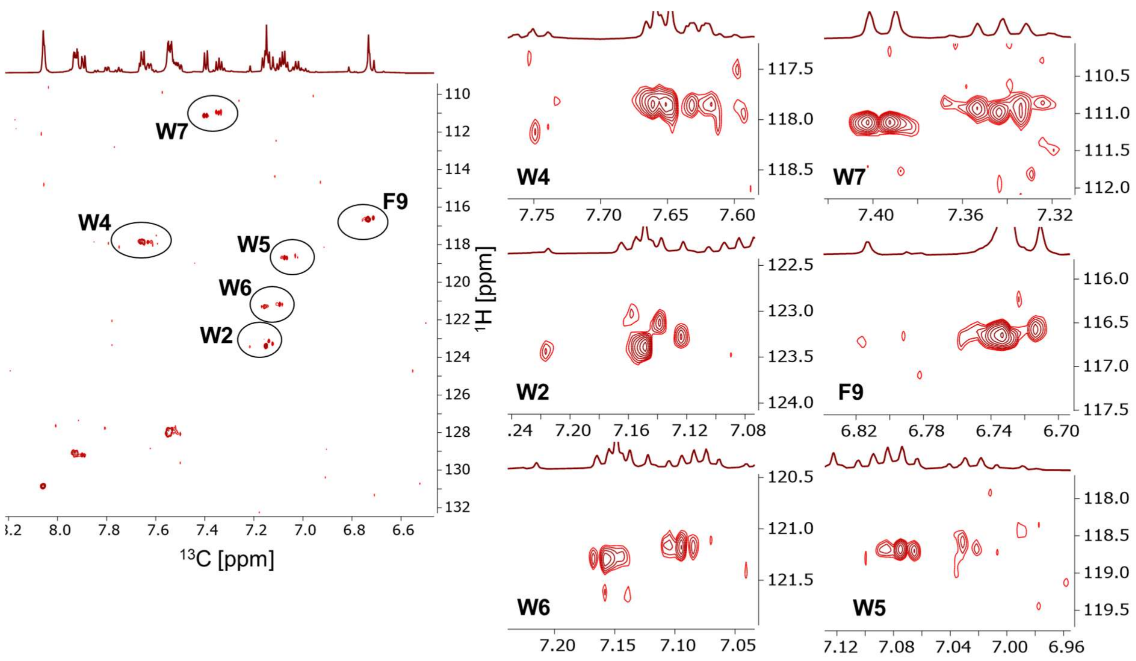

Figure S7. High resolution ^{13}C-^{1}H HSQC spectra 700 MHz: Aromatic part of 3Pro-N*n*Bu dyads ~10 mM in MeOD-d_4 at 293 K.

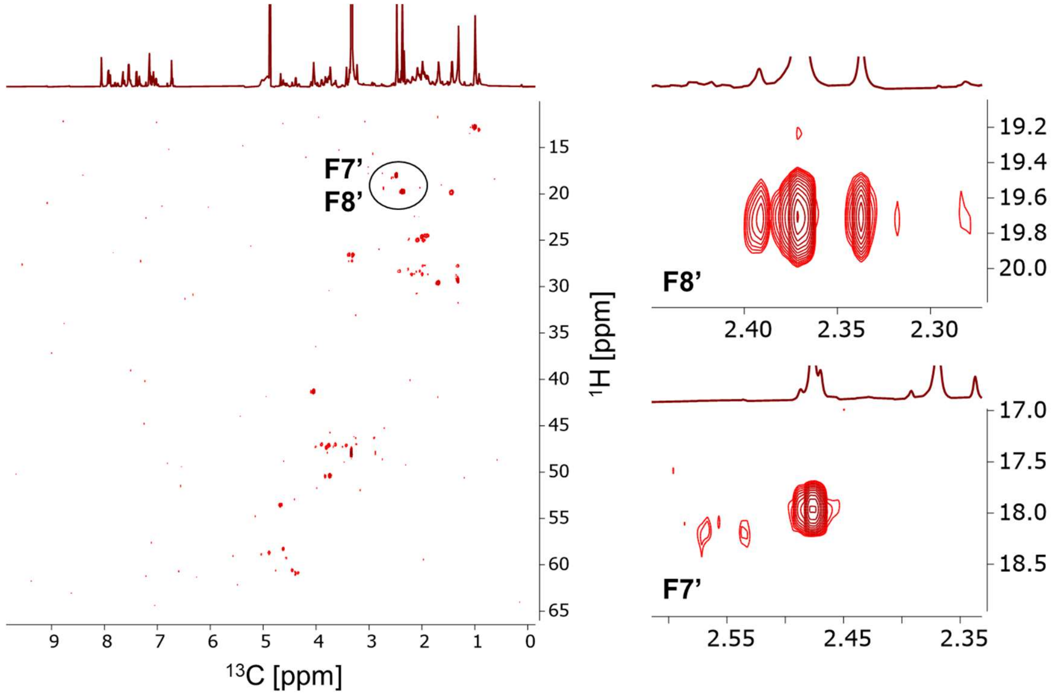

Figure S8. High resolution ^{13}C-^{1}H HSQC spectra @700 MHz: Aliphatic part of 3Pro-N*n*Bu dyads ~10 mM in MeOD-d_4 at 293 K.

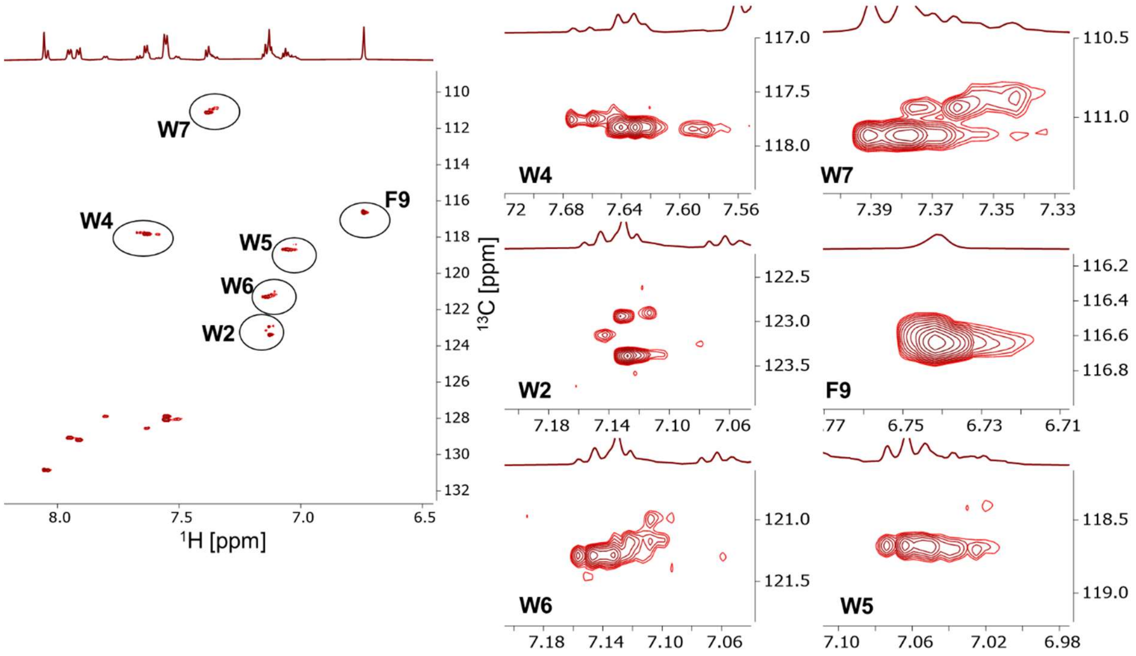

Figure S9. High resolution ^{13}C-^{1}H HSQC spectra @700 MHz: Aromatic part of 6Pro-N*n*Bu dyads ~10 mM in MeOD-d_4 at 293 K.

3. Diffusion ordered spectrosopy

Diffusion ordered spectroscopy (DOSY) and relaxation time measurements were performed for individual protons in a series of dyads. Two representative dyads, 3Pro-N*n*Bu and 9Pro-N*n*Bu (895 Da and 1478 Da, respectively), were selected to illustrate the effect of molecular size. It is known that the diffusion coefficient of molecules in solution depends on their size, as described by the Stokes-Einstein equation, which states that it is inversely proportional to the hydrodynamic radius of the molecule[9]. The results of diffusion coefficient measurements based on the intensities of the flavin methyl protons F7′ and F8′ of the two dyads are shown in Figure S13**B**. The diffusion coefficients for the two dyads were calculated using the SEGWE program, which employs the Stokes-Einstein-Gierer-Wirtz method, as described previously[10]. Notably, there is a good agreement between experimental and calculated data, as well as consistency of the diffusion coefficient with the molecular size: larger molecules exhibit lower diffusion coefficients. Thus, the analysis of diffusion coefficients can be used to identify minor conformers, which in high-resolution NMR spectra at 700 MHz display different chemical shifts (see Figure S13**A**).

Both the total signal intensities of the $F7'_{all}$ and $F8'_{all}$ peaks for the dyads, as well as individual peak contributions, were analyzed by fitting the NMR spectra with a set of Lorentzian lines. The signals corresponding to the minor conformer "m" (signals $F7'_{m}$ and $F8'_{m}$) in the 3Pro-N*n*Bu dyad exhibit an increased diffusion coefficient, indicating a more compact molecular structure in solution. In the case of the 9Pro-N*n*Bu dyad, the situation appears to be the opposite, as only two conformers are observed in the spectrum, see Figure S13**A**. The diffusion coefficient of the minor conformer labeled "m" is lower than that of the major conformer, see Figure S13**B**. However, it should be noted that reliability of this result is limited due to significant signal overlap between the two conformers.

4. Relaxation dispersion

The relaxation dispersion method was used to investigate molecular mobility, specifically the correlation times of rotational motions of molecules in solution. This characteristic depends on the size of the molecule, temperature, and solvent properties, and plays an important role in influencing spin relaxation times. Relaxation dispersion determines whether the molecular motion is slow or fast compared to the Larmor frequency. Field-cycling NMR relaxometry is a versatile technique for studying molecular mobility and can be applied to a wide range of systems, including molecules in solutions, polymers, molecular and liquid crystals, as well as biomolecules[11–13].

Relaxation dispersion measurements, i.e., the dependence of the relaxation rate R_1 on the magnetic field or the Larmor frequency of the nuclei, allow the determination of the rotational correlation time. We have measured T_1 relaxation rates over a range of magnetic fields from 0.1 T to 16.4 T. The dependence of the longitudinal relaxation rate on the magnetic field was plotted and fitted as:

$$R_1(B_0) = R_1^{inf} + \frac{A}{1+(\gamma_H B_0 \tau_c)^2}, \tag{S1}$$

where R_1 is the field dependent longitudinal relaxation rate, B_0 is the magnetic field, γ_H is the proton gyromagnetic ratio, τ_c is the rotational correlation time. This expression represents the simplest relaxation model [13,14], in which fluctuations of local magnetic fields are described by a single parameter τ_c representing the overall molecular tumbling (a detailed analysis is provided in the SI). Analysis of the magnetic field dependence of the relaxation rates for the F methyl protons enables the determination of the rotational correlation times characterizing the global motion of the molecule.

In the field-cycling experiments involving sample shuttling along the magnetic field B_0, the spectral resolution was lower compared to the stationary DOSY experiments. Consequently, reliable integration or deconvolution of individual signals was not always feasible (evidence by the outlier corresponding to the F7′$_{m'}$ signal of the 3Pro dyad). For this reason, only the F8′$_{m'}$, F8′, and F8′$_{m}$ signals were employed in the analysis, as these resonances are well resolved and remain non-overlapping even under field-cycling conditions.

Consistent with the results obtained from diffusion measurements, minor "m" conformers exhibit shorter rotational correlation times, indicating a more compact structure compared to the major conformers (see Figure S13**C**). For the 9Pro-N*n*Bu dyad, it can be assumed that the "m" conformer has a more extended structure, reflected in a longer correlation time and a slight decrease in the diffusion coefficient compared to the major conformer. Notably, in the 3Pro-N*n*Bu molecule, the F8′$_{m}$ signal (on the right side) exhibits the strongest photo-CIDNP polarization in magnetic fields of 10–20 mT (see **Error! Reference source not found.** main text), where the J – mechanism of CIDNP formation is predominant in the biradical.

In conclusion, the minor signals labeled "m" observed for the 3Pro-N*n*Bu dyad are attributed to a conformers with a smaller size and a more compact spatial structure. These conformers are proposed to contain one or more *cis*–configured proline amide bonds at several positions along proline residues, but their exact number and locations cannot be determined. In these *cis*-

containing conformers, the probability of biradical recombination is higher than in the PPII helix composed exclusively of *trans*–proline residues.

4.1 Signal processing

The analysis of the NMR spectra was carried out using the MNOVA software (Mestrelab). For this purpose, the pseudo-2D spectra for each magnetic field were loaded into the program. Then, the spectra were phased and aligned according to the narrowest peak in the spectrum. After that, all the pseudo-2D spectra were combined into a single stack, and batch integration of the proton signals F8′ and F7′ was performed. Figure S10 shows the stacked spectra from the relaxation dispersion experiment for the 3Pro-N*n*Bu and 9Pro-N*n*Bu samples. The integration limits are indicated.

The obtained integral values (intensities) as a function of the delay time at all measured magnetic fields were analysed using Origin (OriginLab). The data for each magnetic field were fitted with a decaying exponential function of the form

$$y = A_1 * exp(-R_1 * x) + y_0. \qquad \text{(S2)}$$

Examples of fittings for different signals at various fields are shown in Figure S11.

The dependence of the relaxation time on the magnetic field was then plotted and fitted using equation

$$R_1(B_0) = R_1^{inf} + \frac{A}{1+(\gamma_H B_0 \tau_c)^2}, \qquad \text{(S3)}$$

derived from the model of nuclear relaxation due to fluctuations of the local magnetic fields [11]. Where γ_H – proton gyromagnetic ratio equals $267.5 \cdot 10^6\ s^{-1}T^{-1}$, B_0 – magnetic field in T, τ_c – rotational correlation time in s, R_1^{inf} – relaxation rate at very high magnetic field. A is the fitting parameter, related to the relaxation rate at low field. The fitting considered the error associated with each R_1 value, meaning that data obtained at higher magnetic fields with smaller errors were given greater weight. As a result, the rotational correlation times, τ_c, for the protons F7′ and F8′ of the two dyads (3Pro-N*n*Bu and 9Pro-N*n*Bu) and their conformers were determined (Table S 2). Examples of the fitting nuclear magnetic relaxation dispersion curves are shown in Figure S12.

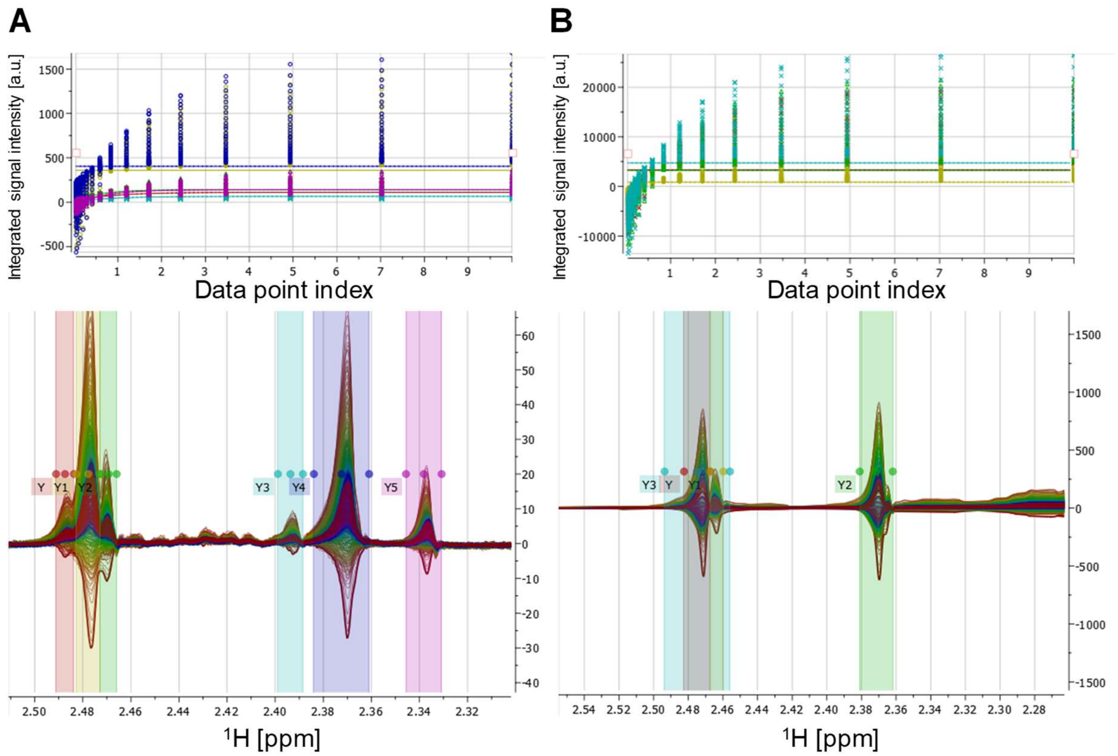

Figure S10. NMR data used for relaxation analysis of 3Pro-N*n*Bu **A** and 9Pro-N*n*Bu **B** measured in magnetic fields ranging from 0.1 to 9.4 T. Top panels show integrated signal intensities as a function of the recovery delay obtained from inversion-recovery experiments. Bottom panels display the corresponding ^{1}H NMR spectra in the F7′ and F8′ spectral regions. Shaded areas indicate the integration limits used for extracting the relaxation kinetics of the resolved F7′ and F8′ signals.

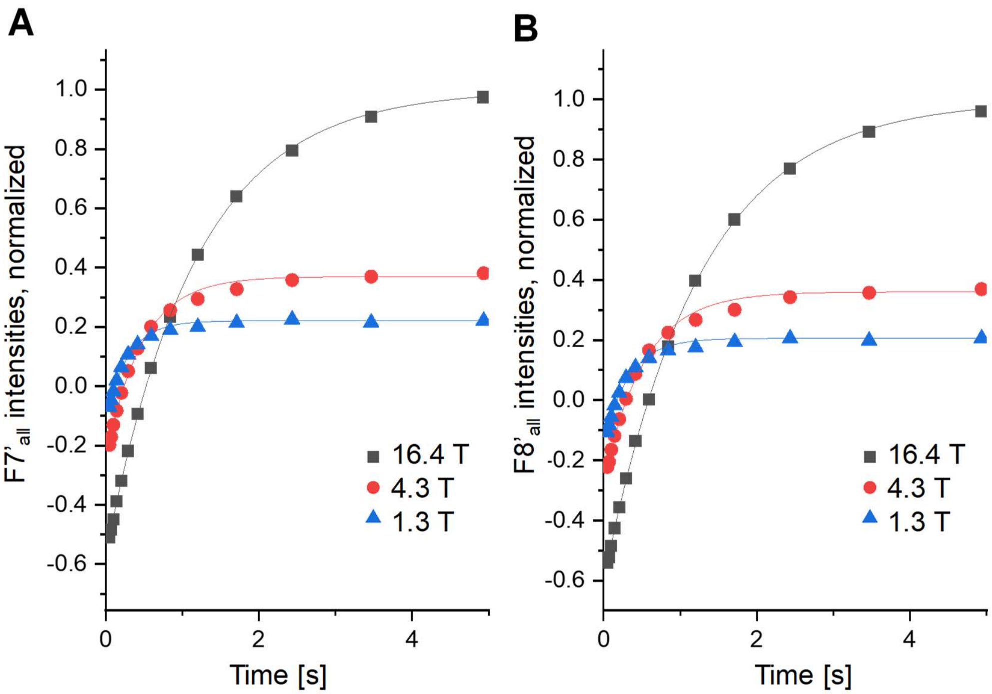

Figure S11. Longitudinal relaxation kinetics of the F7′$_{all}$ **A** and F8′$_{all}$ **B** signals of 9Pro-N*n*Bu measured at magnetic fields B_0 = 16.4, 4.3, and 1.3 T. Symbols represent experimental inversion-recovery data acquired at the indicated magnetic fields, and solid lines correspond to fits using a mono-exponential relaxation model. For F7′$_{all}$, the fitted longitudinal relaxation times are T_1 = 1.16 ± 0.01 s (16.4 T), 0.47 ± 0.02 s (4.3 T), and 0.29 ± 0.02 s (1.3 T). For F8′$_{all}$, the corresponding values are T_1 = 1.24 ± 0.01 s (16.4 T), 0.53 ± 0.03 s (4.3 T), and 0.32 ± 0.02 s (1.3 T).

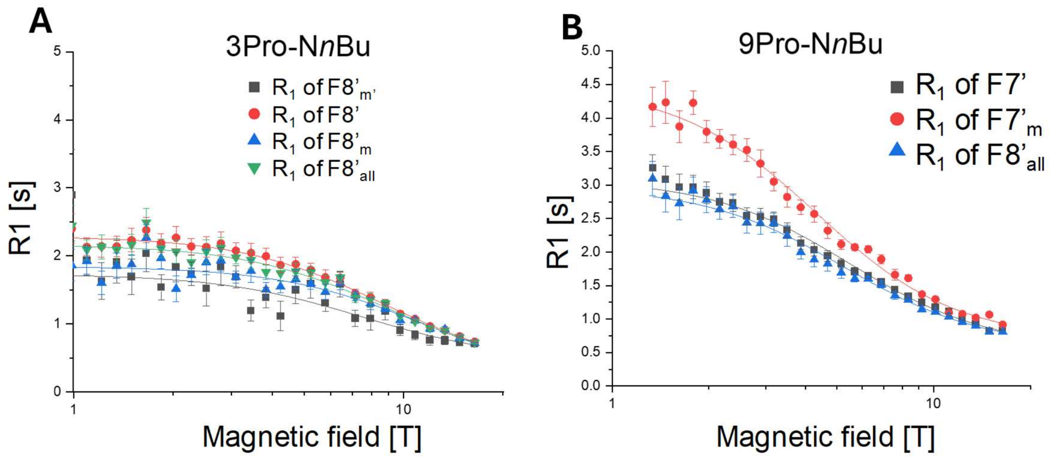

Figure S12. Magnetic-field dependence of the longitudinal relaxation rates (R_1) for nuclei **A** $F8'_{m'}$ ($\boldsymbol{R_0}$=0.476±0.083; A=1.256±0.085), F8′ ($\boldsymbol{R_0}$=0.304±0.030; A=1.985±0.030), $F8'_{m}$ ($\boldsymbol{R_0}$=0.183±0.103; A=1.663±0.088), and $F8'_{all}$ ($\boldsymbol{R_0}$=0.307±0.049; A=1.256±0.085) in 3Pro-N*n*Bu and **B** F7′ ($\boldsymbol{R_0}$=0.566±0.01; A=2.519±0.07), $F7'_{m}$ ($\boldsymbol{R_0}$=0.691±0.03; A=3.780±0.014), and $F8'_{all}$ ($\boldsymbol{R_0}$=0.580±0.01; A=2.391±0.078) in 9Pro-N*n*Bu. Symbols represent experimental data, solid lines correspond to fits using the model of local fluctuating magnetic fields.

Table S2. Rotational correlation times of the dyads 3Pro-N*n*Bu and 9Pro-N*n*Bu and their conformers. For the assignment, see Figure S13.

Dyad	Proton	τ_c, ns
3Pro-N*n*Bu	$F7'_{all}$	0.41±0.01
	$F7'_{m'}$	0.10±0.08
	$F7'$	0.44±0.01
	$F7'_{m}$	0.43±0.07
3Pro-N*n*Bu	$F8'_{all}$	0.42±0.02
	$F8'_{m'}$	0.50±0.08
	$F8'$	0.43±0.01
	$F8'_{m}$	0.33±0.03
9Pro-N*n*Bu	$F7'_{all}$	0.69±0.03
	$F7'$	0.68±0.02
	$F7'_{m}$	0.87±0.04
9Pro-N*n*Bu	$F8'_{all}$	0.71±0.02

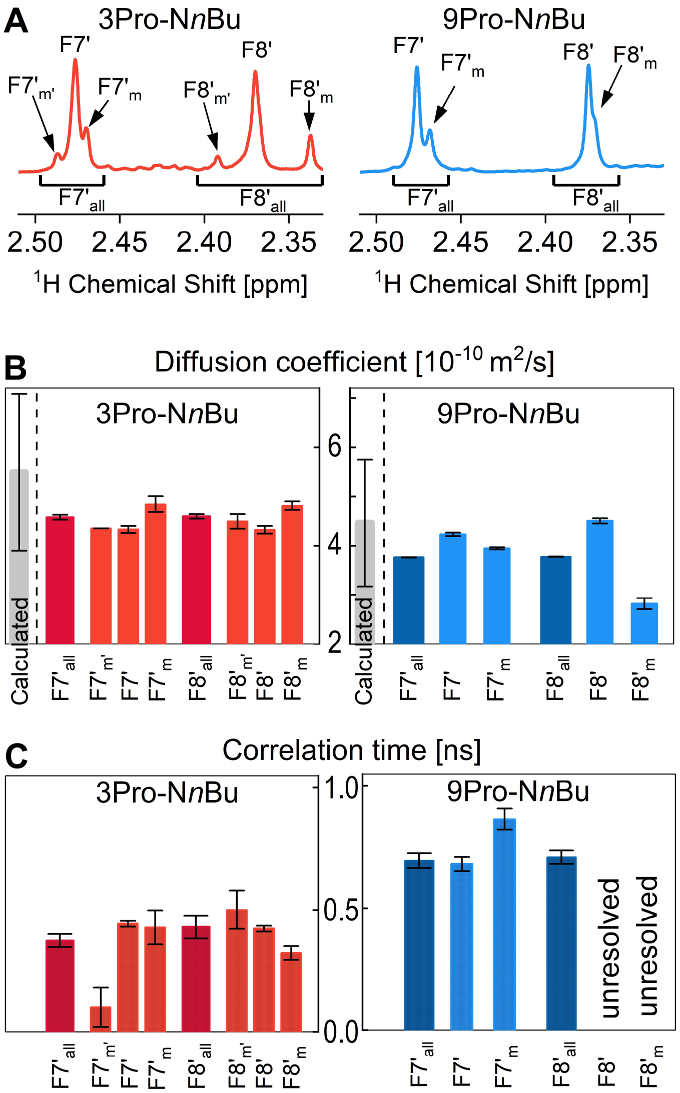

Figure S13. A Methyl region of the 700 MHz ^{1}H NMR spectra of 3Pro-N*n*Bu and 9Pro-N*n*Bu in MeOD-*d*$_4$ at ~10 mM concentration. B Diffusion coefficients recorded for the 3Pro-N*n*Bu and 9Pro-N*n*Bu dyads measured by DOSY for the combined flavin signals (F7′$_{all}$, F8′$_{all}$) and for the resolved minor conformer signals (F7′$_{m}$, F8′$_{m}$). The calculated diffusion coefficients (using the SEGWE program) are shown in blue. C Rotational correlation times determined from nuclear magnetic relaxation dispersion (NMRD) for the 3Pro-N*n*Bu and 9Pro-N*n*Bu dyads.

5. Time-Resolved photo-CIDNP

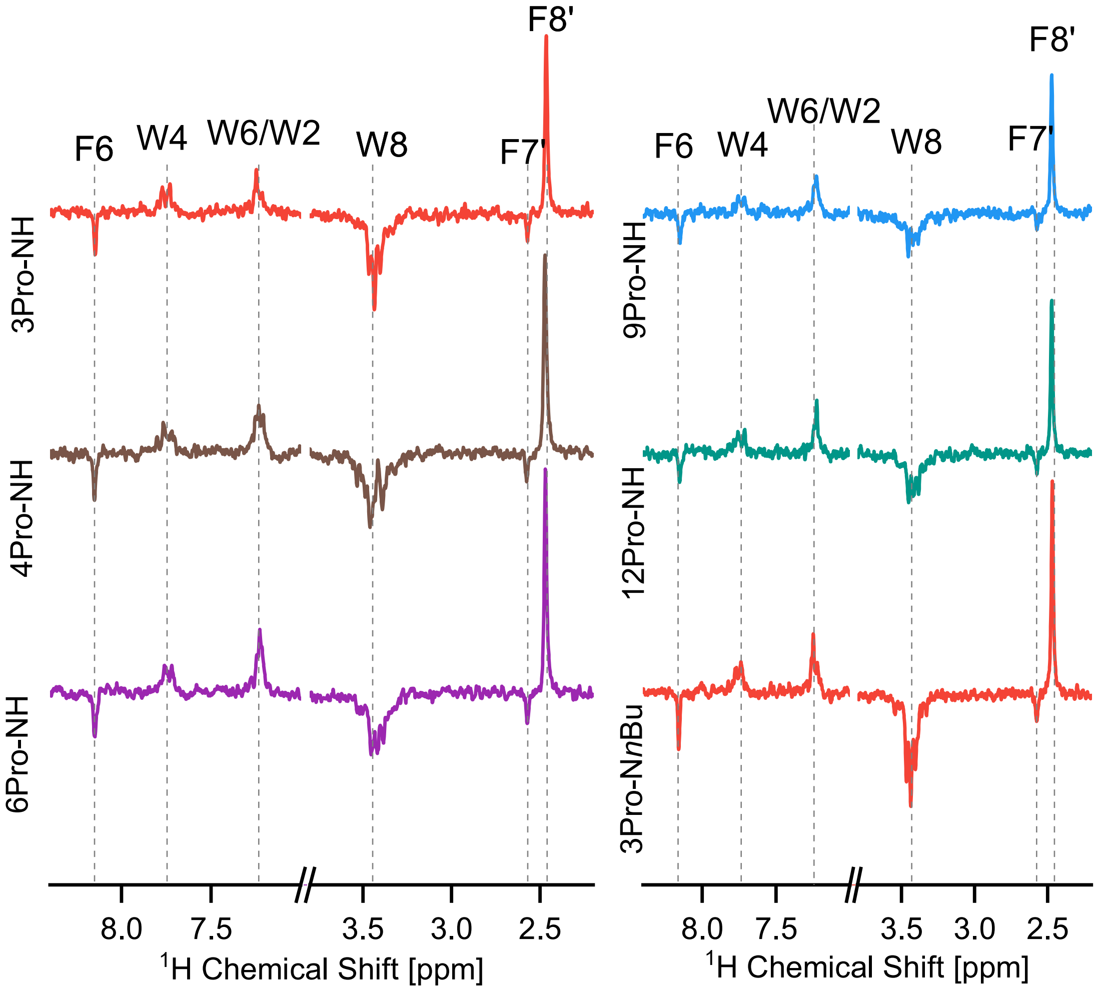

Figure S14. 200 MHz ^{1}H photo-CIDNP spectra of W-(Pro)$_n$-F-NH and W-(Pro)$_n$-FN*n*Bu conjugates 0.2 mM in MeOD-*d*$_4$, recorded immediately after the laser flash. The RF detection pulse duration was 4 µs. Assignments correspond to tryptophan (W2, W4, W6, W8) and flavin (F6, F7, F8′) protons.

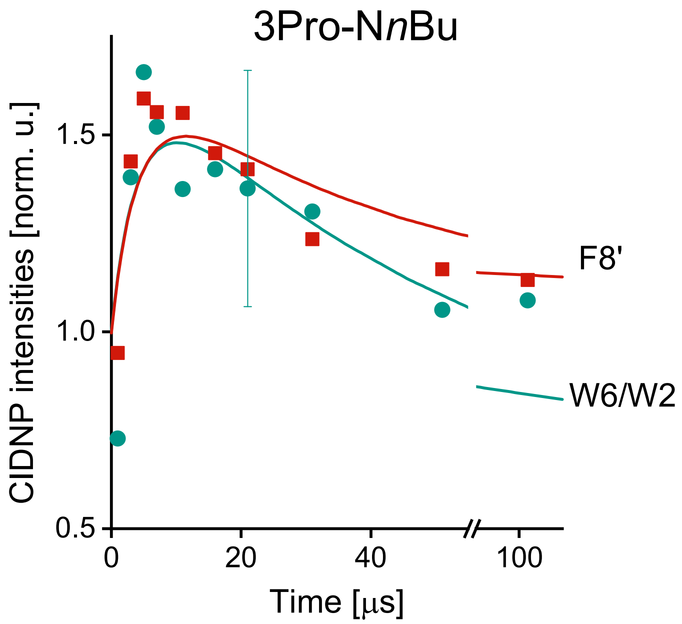

Figure S15. Photo-CIDNP kinetics of 3Pro-N*n*Bu 0.2 mM in MeOD-d_4. CIDNP signal intensities of protons F8′ and W6/W2 are shown as a function of the delay time after excitation with a 308 nm laser (pulse length ≈20 ns). The RF detection pulse duration was 2 μs, time delay values in this figure correspond to the center of the detecting pulse: 1 μs for delay 0 μs, 3 μs for delay 2 μs, etc. Intensities are normalized to the value at the earliest time point.

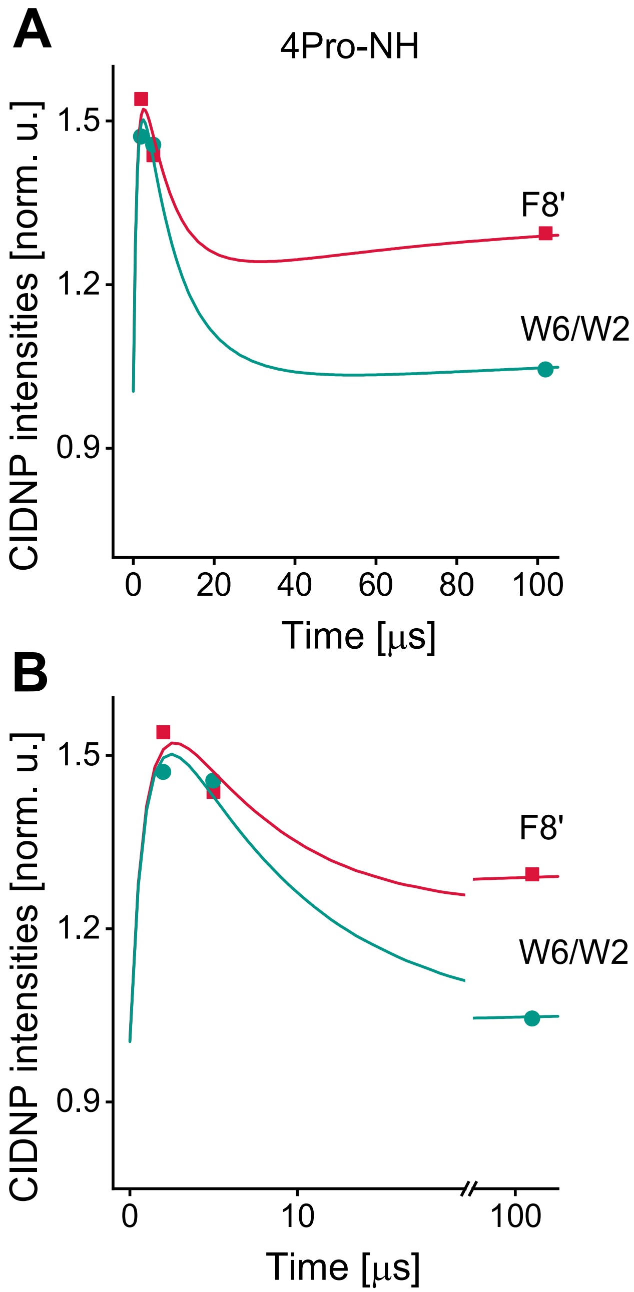

Figure S16. Photo-CIDNP kinetics of 4Pro-NH 0.2 mM in MeOD-d_4. CIDNP signal intensities of protons F8′ and W4/W2 are shown as a function of the delay time after excitation with a 308 nm laser (pulse length ≈20 ns). The RF detection pulse duration was 4 μs. Intensities are normalized to the value at the earliest time point. (A) Time axis without break; (B) time axis with break.

6. Field-cycling photo-CIDNP

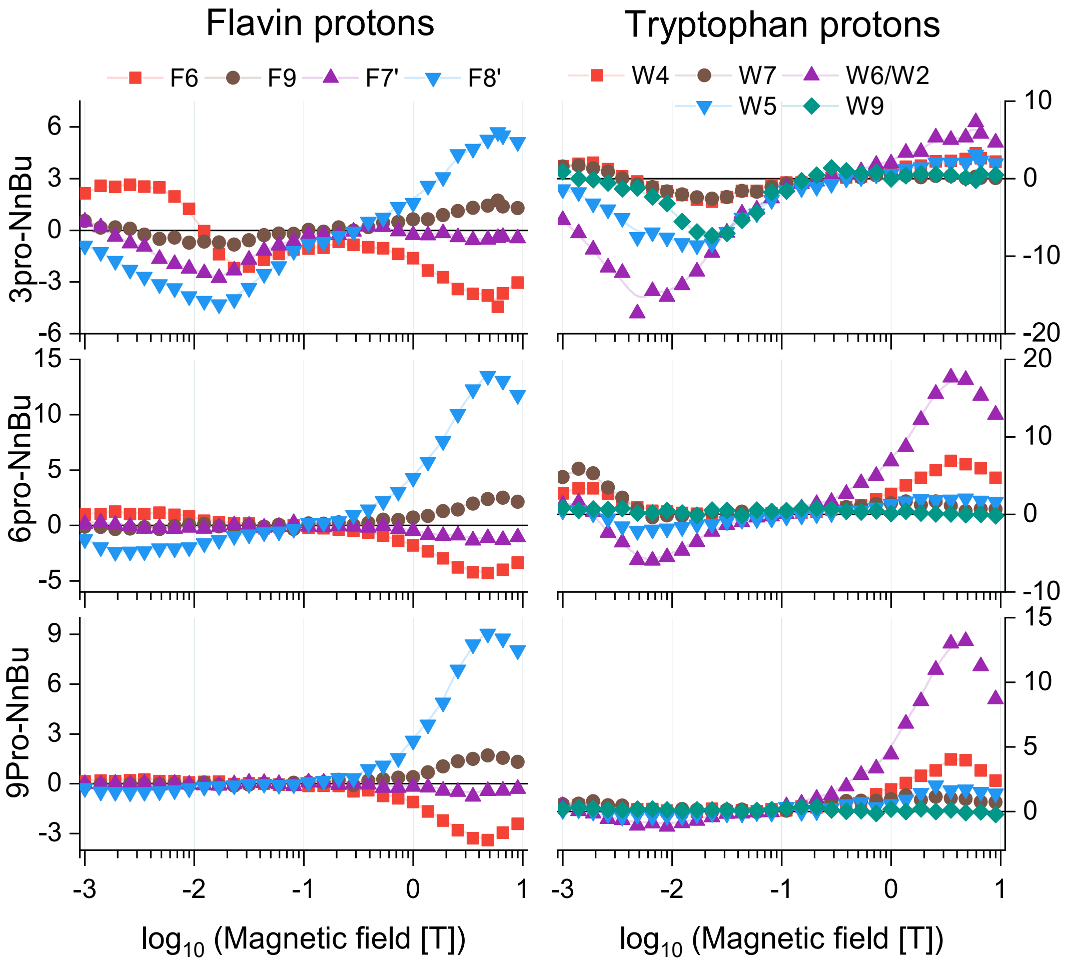

Figure S17. Magnetic field dependence of photo-CIDNP intensities for individual protons in flavin–tryptophan dyads. Each curve shows the polarization amplitude of a specific proton signal as a function of magnetic field, measured upon 450 nm LED irradiation. Left panels: flavin (acceptor) protons F6, F9, F7', and F8′; right panels: tryptophan (donor) protons W4, W7, W6/W2, W5, and W9. Rows correspond to different samples: 3Pro-N*n*Bu, 6Pro-N*n*Bu, and 9Pro-N*n*Bu. Data are plotted versus $\log_{10}$ of the magnetic field (T); lines are guides for the eye.

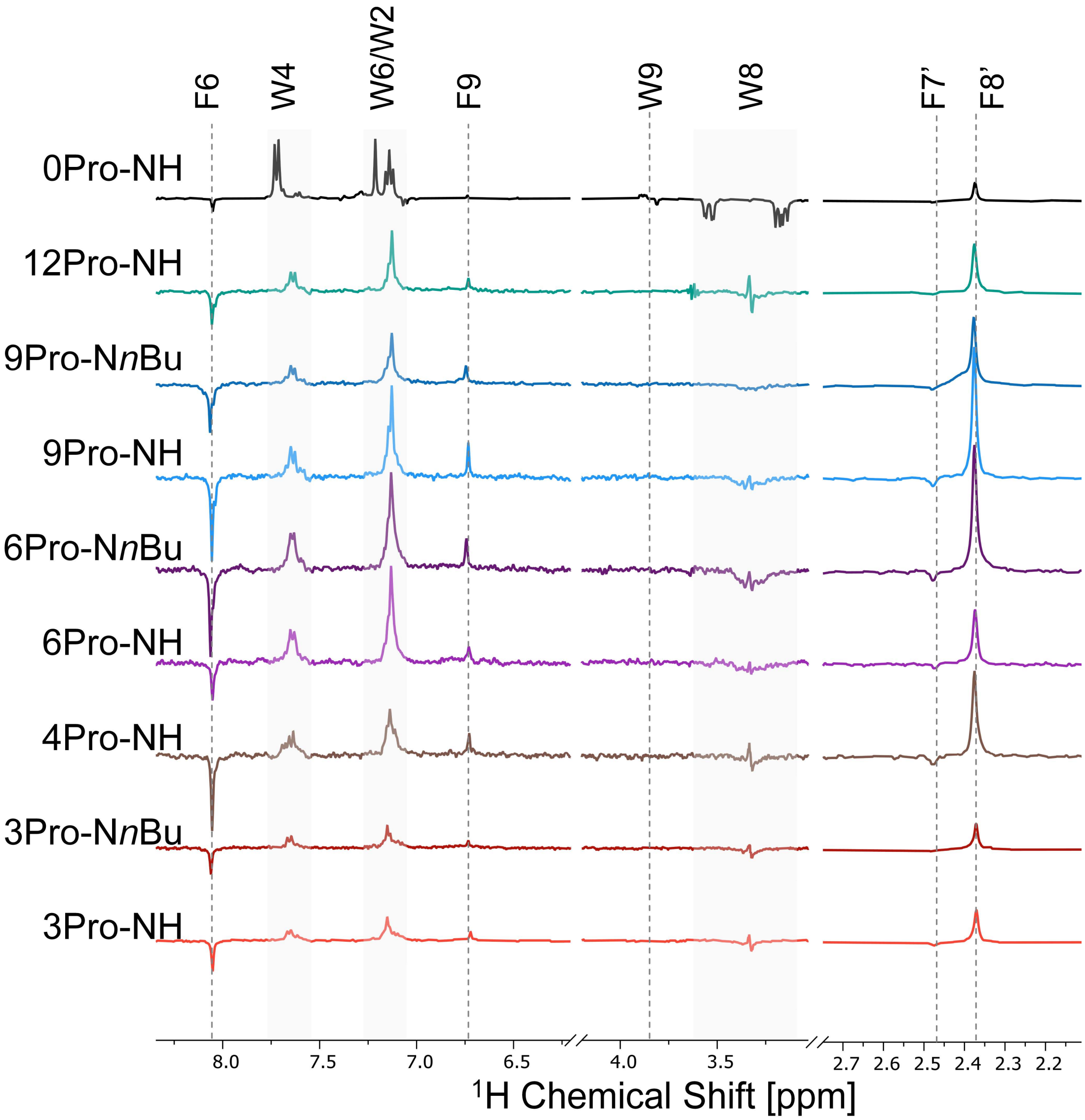

Figure S18. Photo-CIDNP spectra of flavin–tryptophan dyads with polyproline linkers of varying length (3–12 residues) and unconjugated flavin/tryptophan (top trace), recorded at 9 T ~0.2 mM in MeOD-d_4 upon 450 nm LED irradiation. Emissive and absorptive signals correspond to polarized protons of flavin and tryptophan moieties; linker length is indicated for each trace.

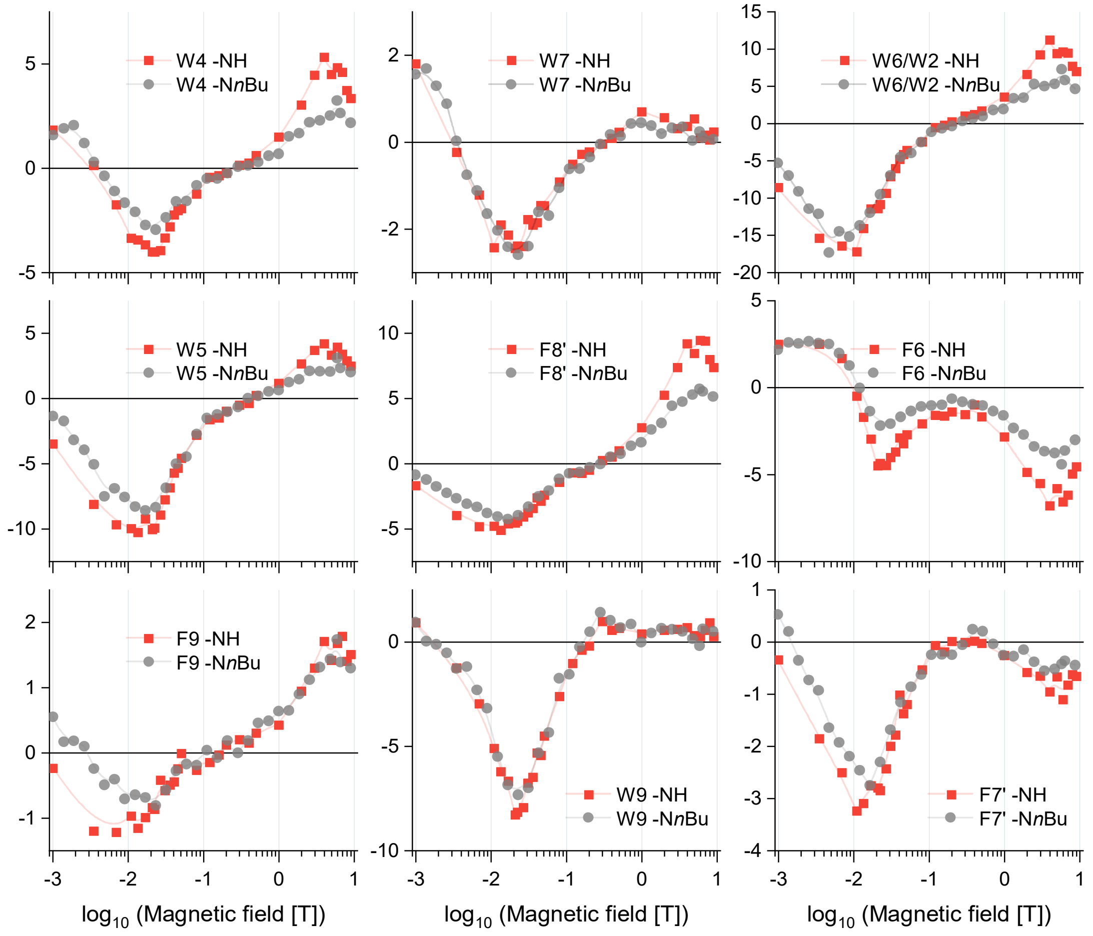

Figure S19. Magnetic field dependence of photo-CIDNP intensities for individual protons in 3Pro-NH and 3Pro-N*n*Bu dyads. Each panel shows the polarization amplitude of a given proton signal (flavin or tryptophan) plotted versus $\log_{10}$ of the magnetic field (T) upon 1 s, 450 nm (20 mW) LED irradiation ~0.2 mM in MeOD-d_4 at 293 K. Data for the two dyads are overlaid for direct comparison. Lines are used for guidance.

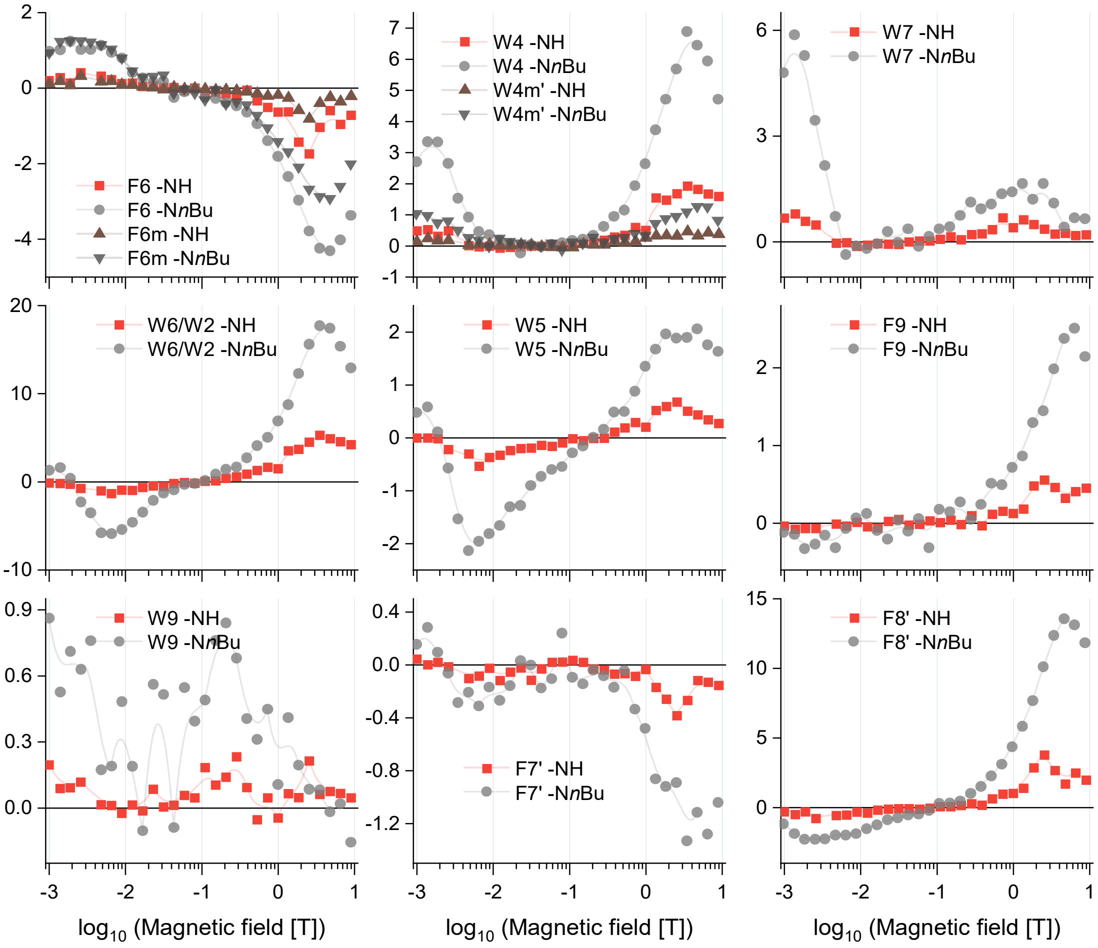

Figure S20. Magnetic field dependence of photo-CIDNP intensities for individual protons in 6Pro-NH and 6Pro-N*n*Bu dyads. Each panel shows the polarization amplitude of a given proton signal (flavin or tryptophan) plotted against the $\log_{10}$ of the magnetic field (T) after 1 s 450 nm (20 mW) LED irradiation ~0.2 mM in MeOD-d_4 at 293 K. Data for the two dyads are overlaid for direct comparison. Lines are used for guidance.

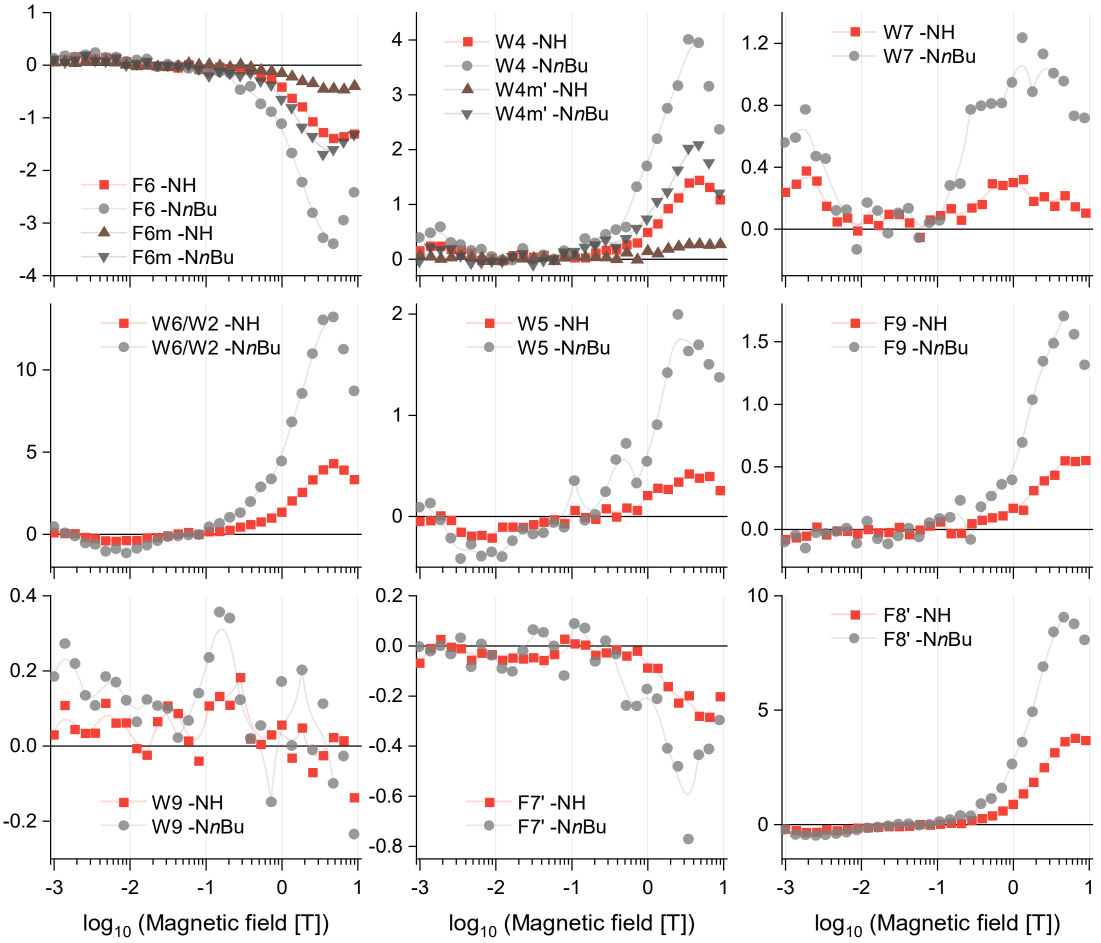

Figure S21. Magnetic field dependence of photo-CIDNP intensities for individual protons in 9Pro-NH and 9Pro-N*n*Bu dyads. Each panel shows the polarization amplitude of a given proton signal (flavin or tryptophan) plotted against the $\log_{10}$ of the magnetic field (T) after 1 s of 450 nm (20 mW) LED irradiation ~0.2 mM in MeOD-d_4 at 293 K. Data for the two dyads are overlaid for direct comparison. Lines are used for guidance.

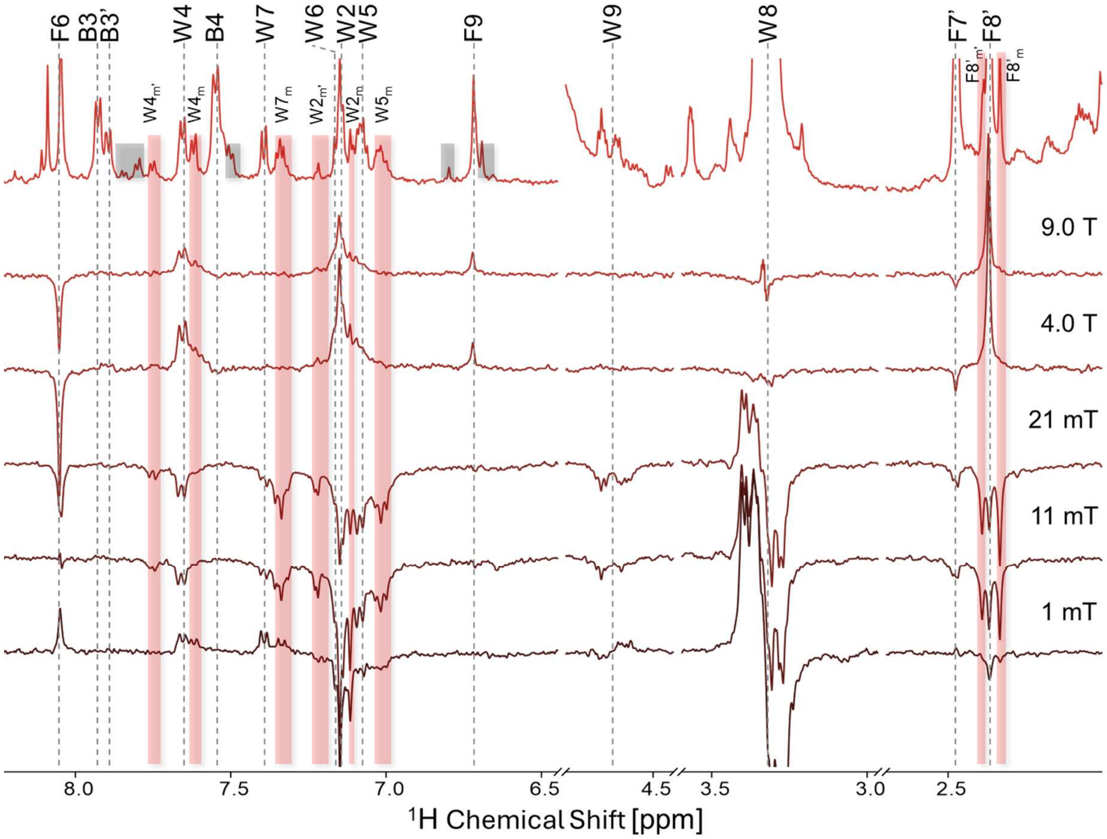

Figure S22. ^{1}H NMR and field-dependent photo-CIDNP spectra of dyad 3pro-NH in MeOD-d_4 at 293 K. Top: conventional ^{1}H NMR spectrum ~10 mM at 14.1 T. Bottom: photo-CIDNP difference spectra at ~0.2 mM upon 1 s, 450 nm (20 mW) irradiation at fields from 9.0 T to 1 mT. Dashed lines mark major conformer signals; black and red shading indicate minor conformers, with red denoting photo-CIDNP-active sites.

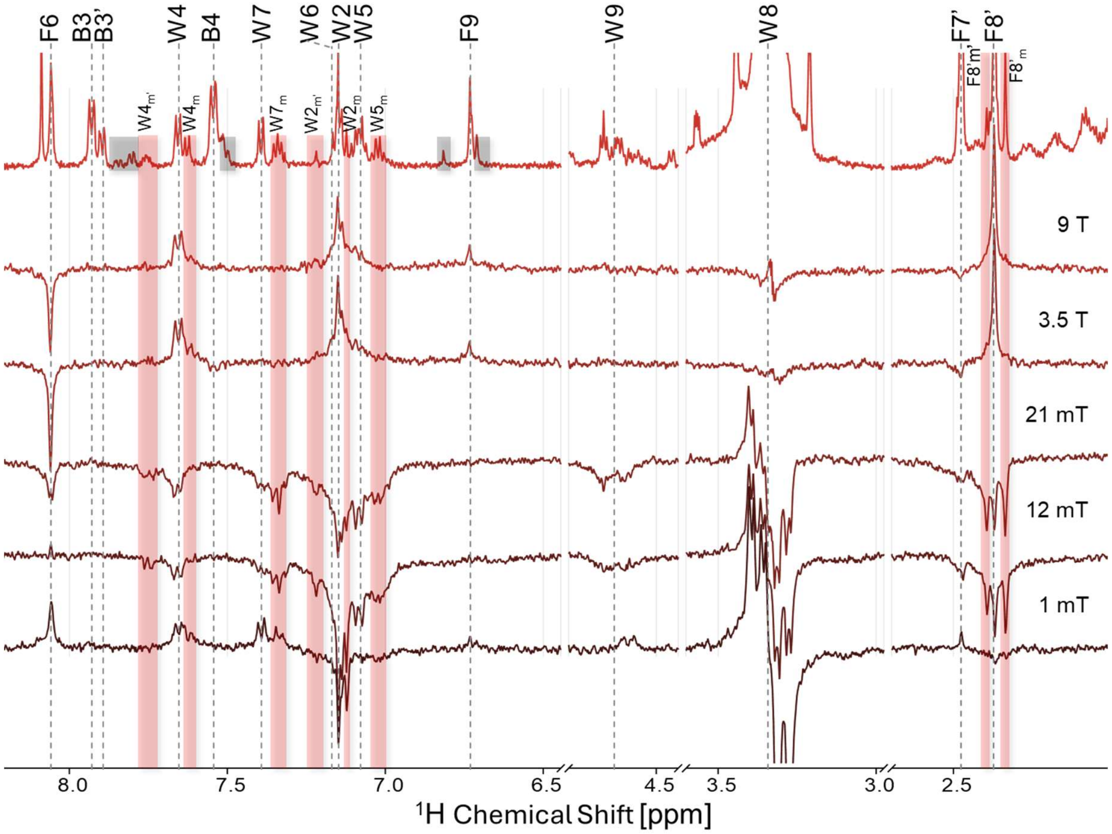

Figure S23. ^{1}H NMR and field-dependent photo-CIDNP spectra of 3Pro-N*n*Bu in MeOD-*d₄* (293 K). Top: conventional ^{1}H NMR spectrum ~10 mM at 14.1 T. Bottom: photo-CIDNP difference spectra at ~0.2 mM upon 1 s irradiation with a 450 nm, 20 mW LED at magnetic fields from 9.0 T to 1 mT. Vertical dashed lines indicate major conformer signals; black and red shading denote minor conformers, with red marking those exhibiting photo-CIDNP effects.

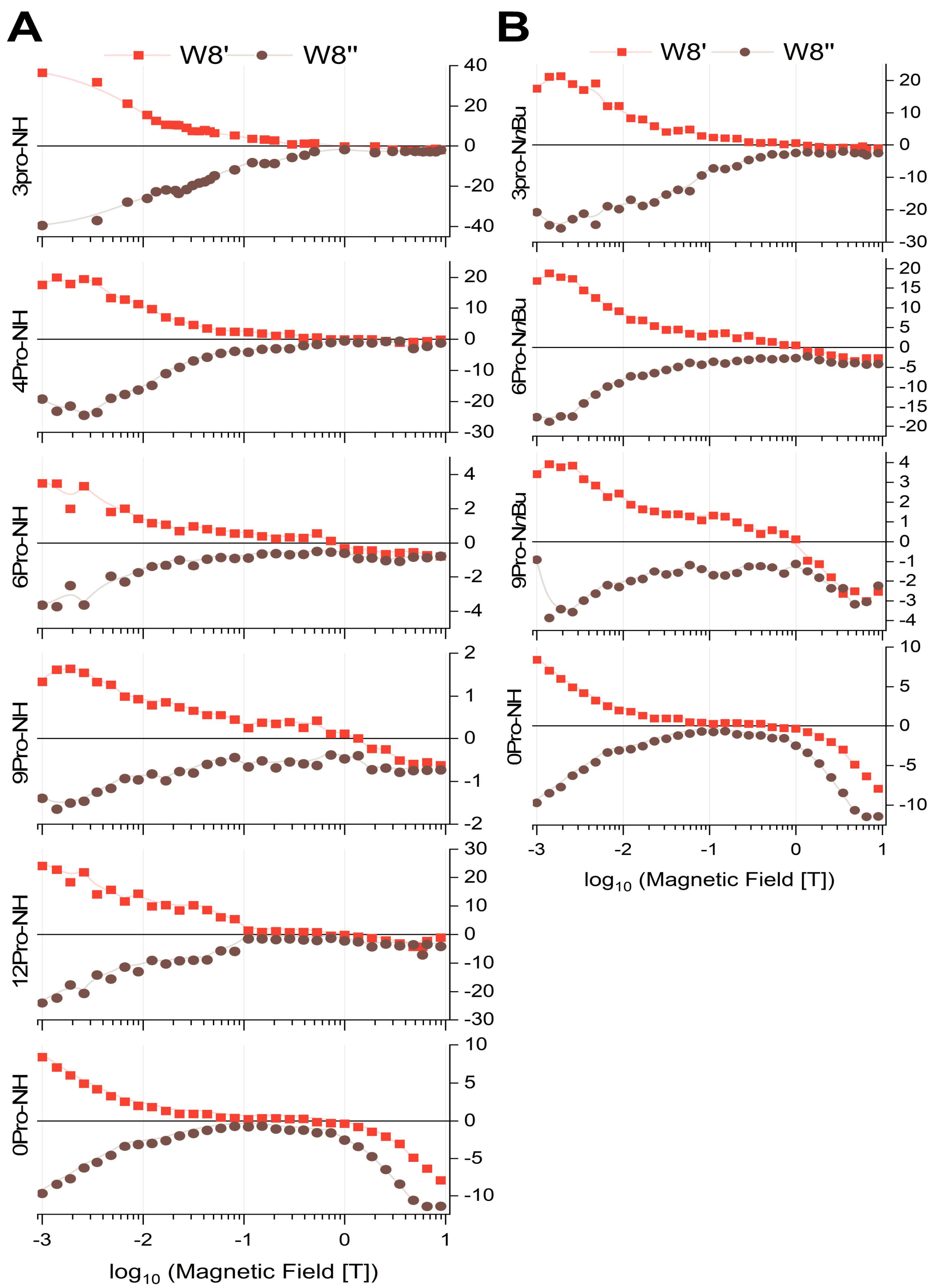

Figure S24. Magnetic field dependence of photo-CIDNP intensities for two protons (W8' and W8'') in flavin–tryptophan dyads with varying linker lengths. Data are shown on the panel **A** for 3Pro-NH, 4Pro-NH, 6Pro-NH, 9Pro-NH, and 12Pro-NH, and on the panel **B** 3Pro-N*n*Bu, 6Pro- N*n*Bu, 9Pro- N*n*Bu and unconjugated flavin and tryptophan (1:1 ratio) in MeOD-*d*4 in concentration of ~0.2 mM at 293 K, upon 1 s irradiation with a 450 nm, 20 mW LED. Polarization amplitudes are plotted versus $\log_{10}$ of the magnetic field (T); lines are guides for the eye.

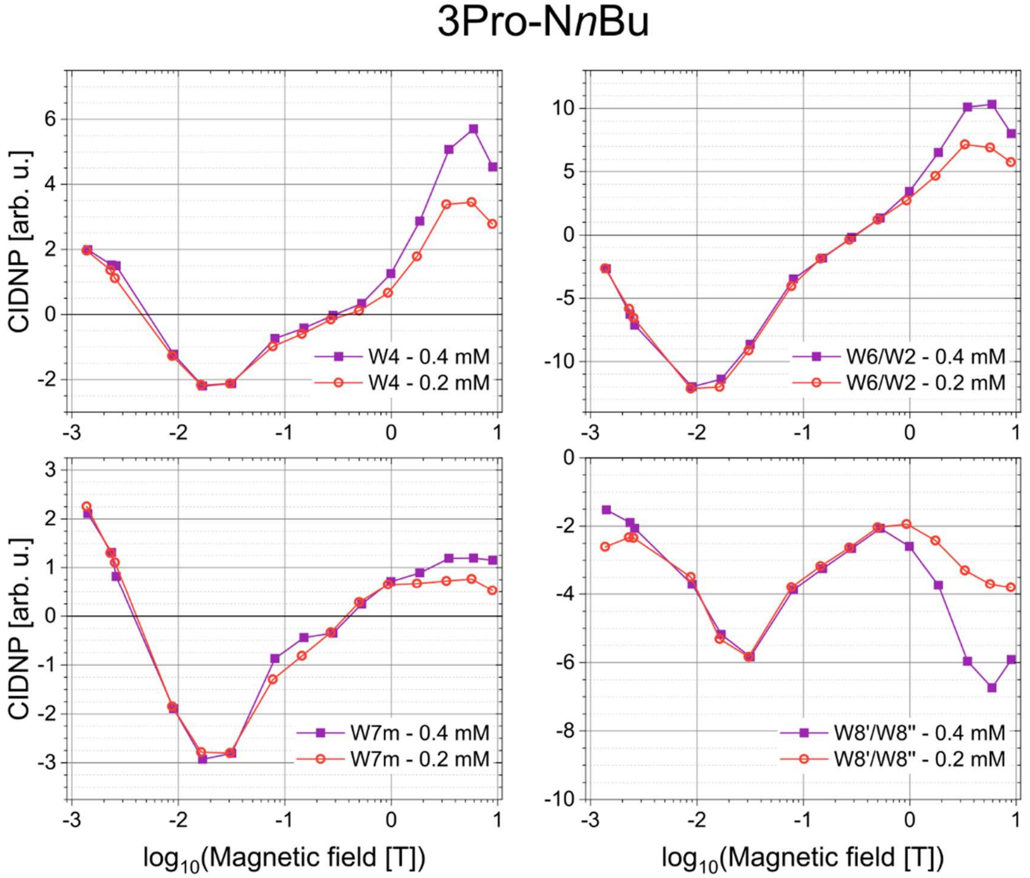

Figure S25. Magnetic field dependence of photo-CIDNP intensities for protons W4, W6/W2, W7m, and total intensity of W8'/W8'' proton pair of two 3Pro-N*n*Bu solutions in MeOD: ~0.4 mM (violet squares) and ~0.2 mM (red open circles), at 293 K, upon 1 s irradiation with a 450 nm, 20 mW LED. Polarization amplitudes are plotted versus $\log_{10}$ of the magnetic field (T). CIDNP amplitudes at ~10 mT were normalized to the same value to facilitate the comparison between lineshapes of CIDNP field dependences.

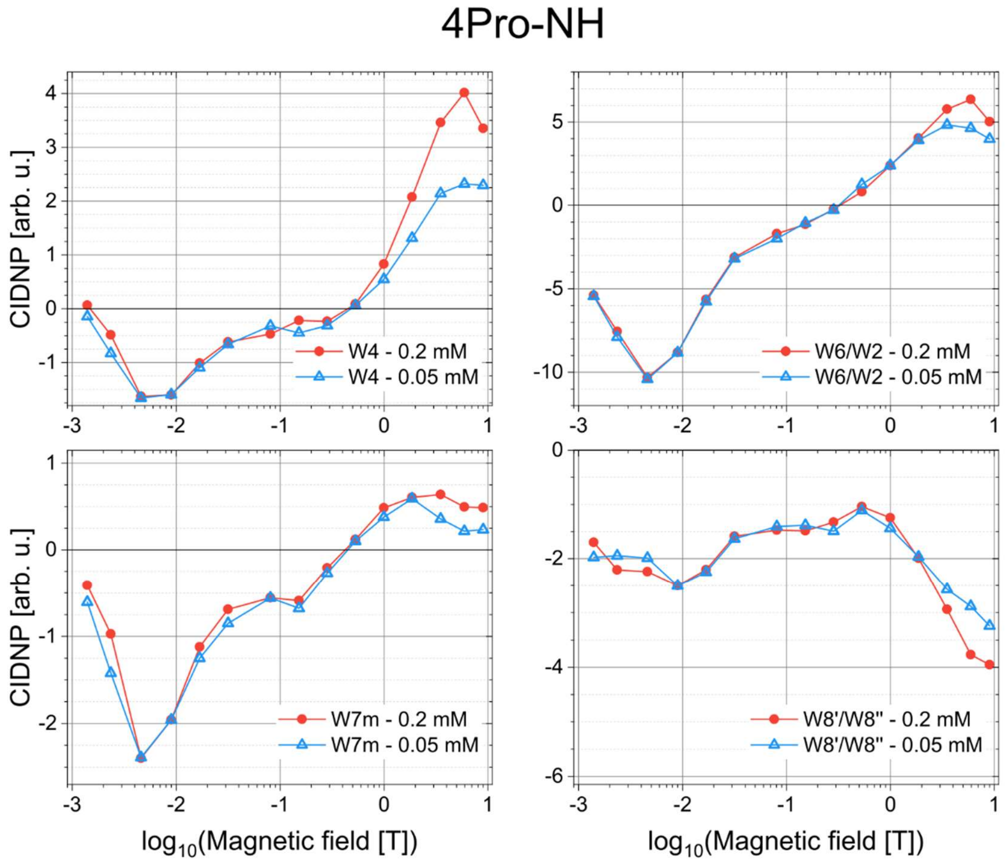

Figure S26. Magnetic field dependence of photo-CIDNP intensities for protons W4, W6/W2, W7m, and total intensity of W8'/W8'' proton pair of two 4Pro-NH solutions in MeOD: ~0.2 mM (red circles) and ~0.05 mM (blue open triangles), at 293 K, upon 1 s irradiation with a 450 nm, 20 mW LED. Polarization amplitudes are plotted versus $\log_{10}$ of the magnetic field (T). CIDNP amplitudes at ~10 mT were normalized to the same value to facilitate the comparison between lineshapes of CIDNP field dependences.

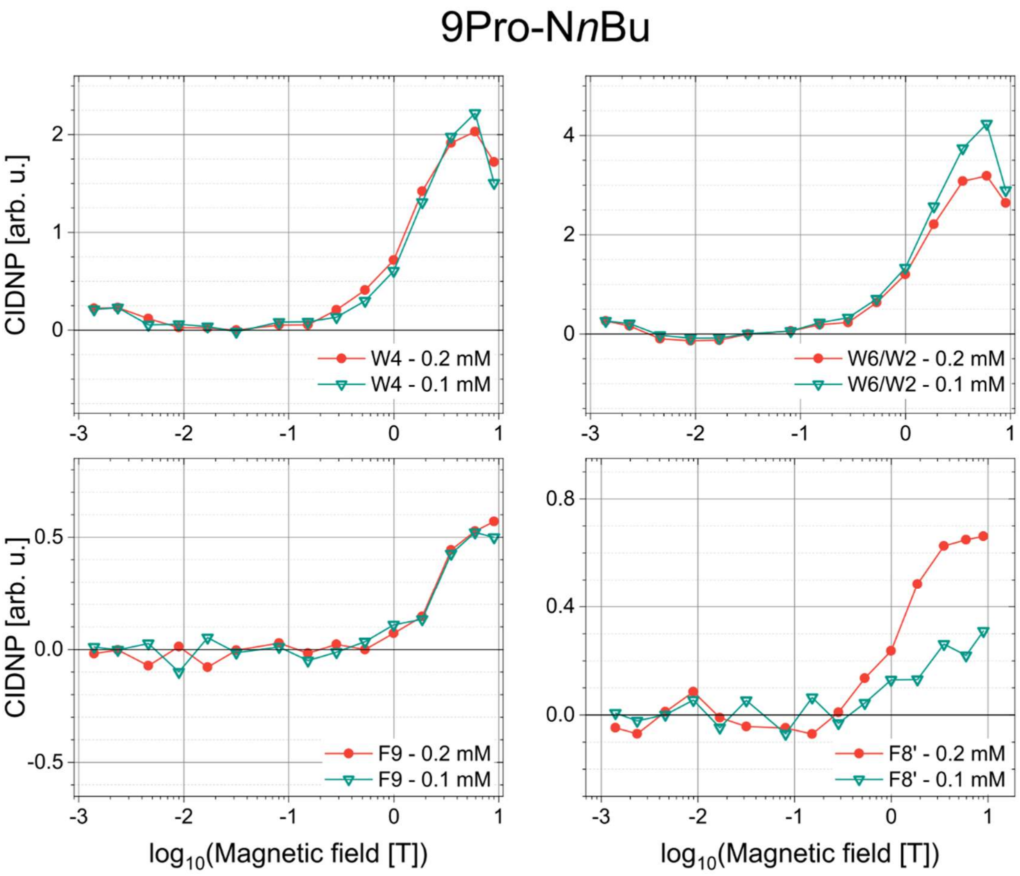

Figure S27. Magnetic field dependence of photo-CIDNP intensities for protons W4, W6/W2, F9, and F8’ of two 9Pro-N*n*Bu solutions in MeOD: ~0.2 mM (red circles) and ~0.05 mM (green open triangles), at 293 K, upon 1 s irradiation with a 450 nm, 20 mW LED. Polarization amplitudes are plotted versus $\log_{10}$ of the magnetic field (T). CIDNP amplitudes at ~2-6 mT were normalized to the same value to facilitate the comparison between lineshapes of CIDNP field dependences.

7. Additional results from molecular dynamics simulations

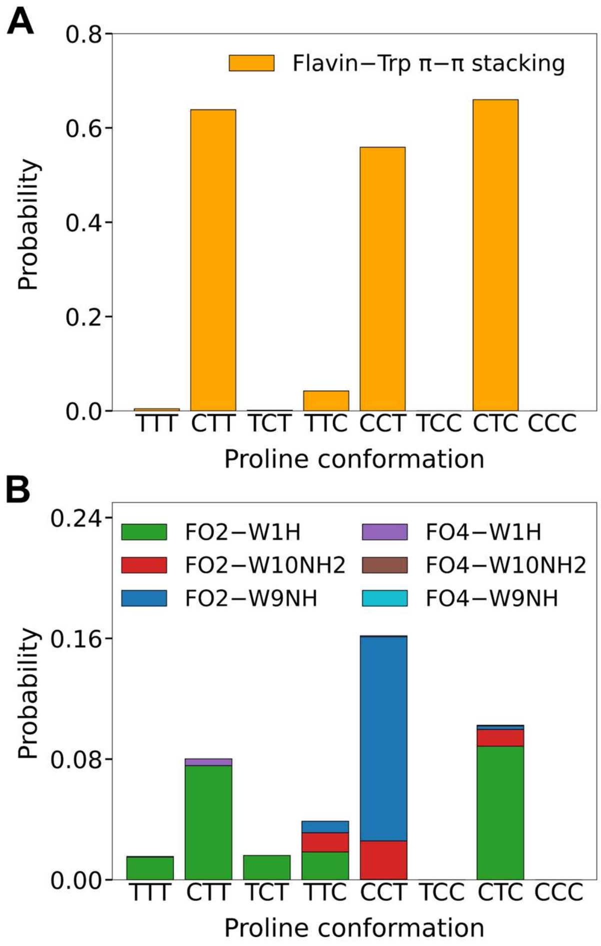

Figure S28. A Conditional probability of observing a π-π stacked flavin-tryptophan arrangement within a given fixed proline-chain configuration in the MD simulations of 3Pro-NH. Values reflect the fraction of frames fulfilling the stacking criteria for each configuration separately. **B** Hydrogen bond formation probability between F and W found in the 3Pro-NH dyads MD simulations for different possible proline conformations. The labels in panel B refer to the oxygen and the hydrogen atoms between which the hydrogen bond is formed. The "T" and "C" labels refer to *trans*– and *cis*–proline conformations, respectively, where numeration starts from the proline closest to the F moiety.

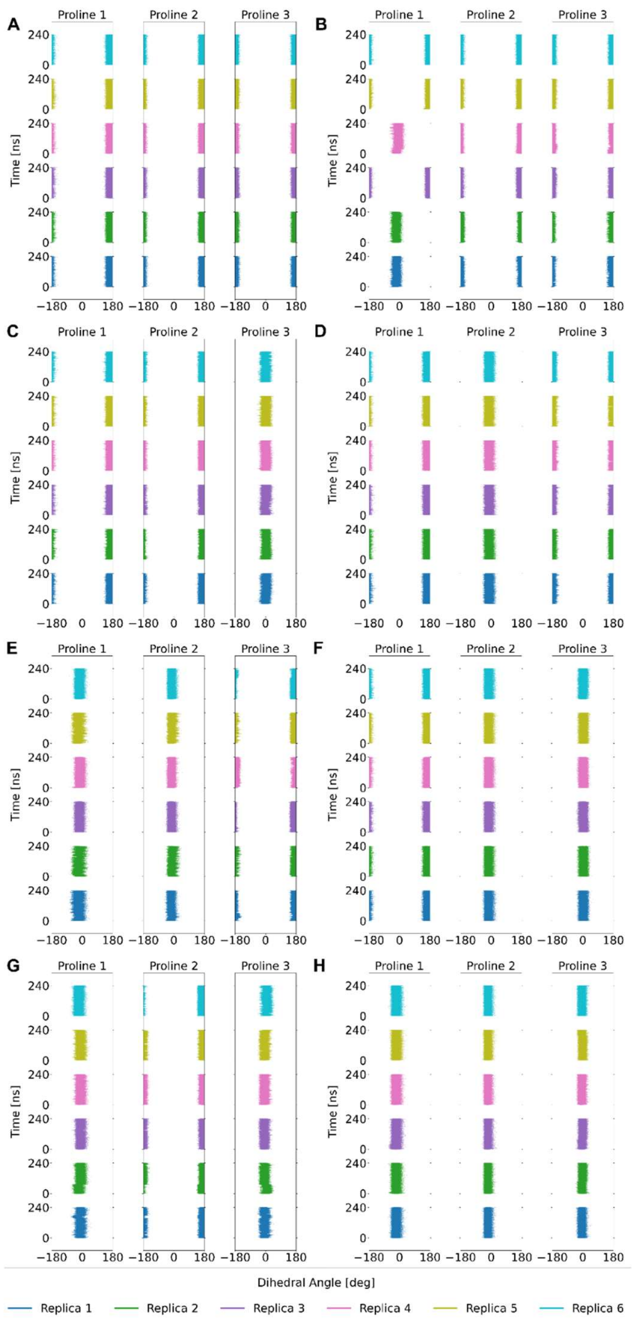

Figure S29. **A** Temporal evolution of the peptide bond dihedral angle **ω** obtained from MD simulations of 3Pro-NH dyad with the *trans-trans-trans* configurations of the prolines. Numbering of prolines starts with the one closest to the flavin. **B** Similar to **A**, but computed for the *cis-trans-trans* proline configurations. **C** Similar to **A**, but computed for the *trans-trans-cis* proline configurations. **D** Similar to **A**, but computed for the *trans-cis-trans* proline configurations. **E** Similar to **A**, but computed for the *cis-cis-trans* proline configurations. **F** Similar to **A**, but computed for the *trans-cis-cis* proline configurations. **G** Similar to **A**, but computed for the *cis-trans-cis* proline configurations. **H** Similar to **A**, but computed for the *cis-cis-cis* proline configurations.

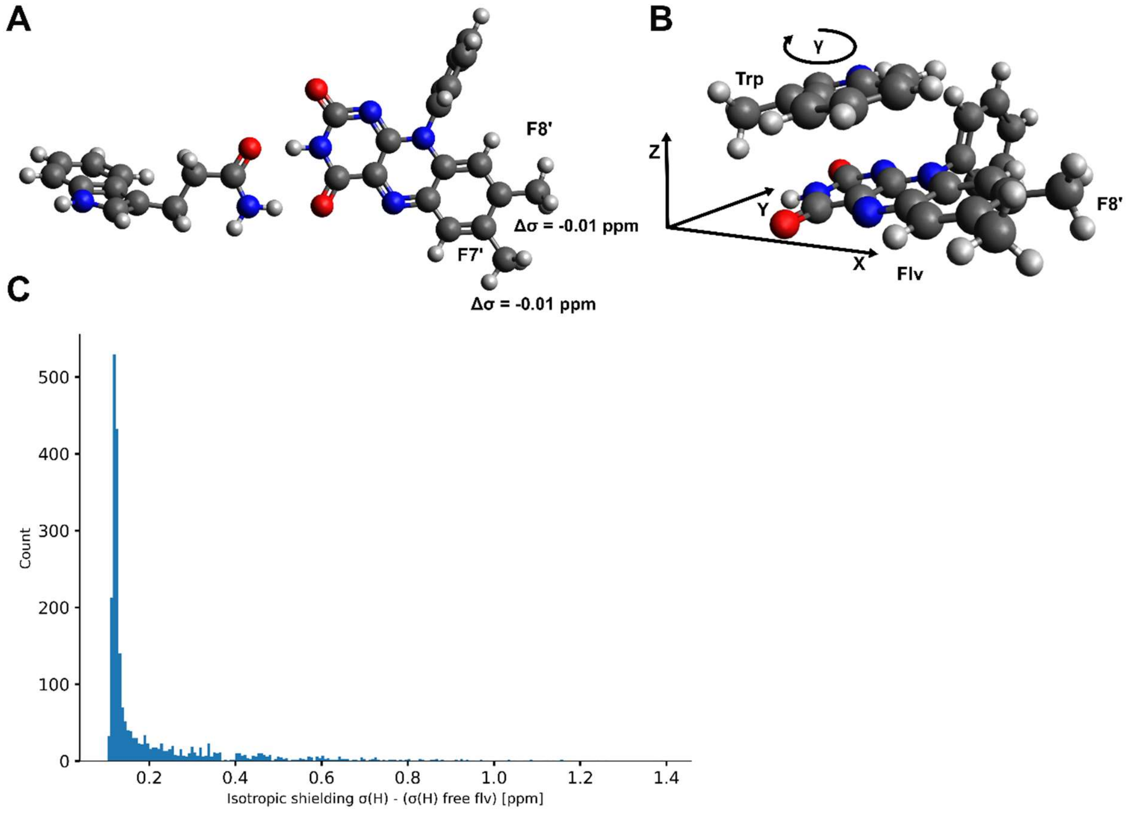

Figure S30. A Geometrically optimized system including a flavin-linker moiety and a tryptophan residue resolving in hydrogen bonding between the amide group of tryptophan and the heteroaromatic ring system of flavin. For both, F7 and F8, small down-shielding effects can be found. **B** Illustration of the used parameter (X, Y, Z, γ) to scan the chemical shielding behaviour due to stacking between tryptophan and flavin. The tryptophan is moved around the X, Y and Z axis with respect to the flavin plane, while additionally the tryptophan is rotated around its aromatic plane axis via the parameter γ. **C** Distribution of calculated isotropic chemical shieldings corresponding to the conformational space sampled in panel B. Each count represents a precise set of (X, Y, Z, γ) values. As can be observed, only up-shielding effects can be found compared to a free flavin-linker system.

8. References

(1) Theiss, T.; Musabirova, G.; Gerhards, L.; Anisimova, I. S.; Zoller, B. G. E.; Nguyen, T. N. H.; Denisov, D.; Schmidt, A.; Panter, S.; Weber, S.; Tegenkamp, C.; Solov'yov, I. A.; Matysik, J.; Gulder, T. Distance-Dependence of Photo-CIDNP in Biomimetic Tryptophan-Flavin Diads. *Angew Chem Int Ed Engl* **2025**, *64* (38), e202510116. DOI: 10.1002/anie.202510116.

(2) Zhukov, I. V.; Kiryutin, A. S.; Yurkovskaya, A. V.; Grishin, Y. A.; Vieth, H.-M.; Ivanov, K. L. Field-cycling NMR experiments in an ultra-wide magnetic field range: relaxation and coherent polarization transfer. *Phys Chem Chem Phys* **2018**, *20* (18), 12396–12405. DOI: 10.1039/C7CP08529J.

(3) Zhukov, I. V.; Kiryutin, A. S.; Panov, M. S.; Fishman, N. N.; Morozova, O. B.; Lukzen, N. N.; Ivanov, K. L.; Vieth, H.-M.; Sagdeev, R. Z.; Yurkovskaya, A. V. Exchange interaction in short-lived flavine adenine dinucleotide biradical in aqueous solution revisited by CIDNP (chemically induced dynamic nuclear polarization) and nuclear magnetic relaxation dispersion. *Magn Reson (Gott)* **2021**, *2* (1), 139–148. DOI: 10.5194/mr-2-139-2021.

(4) Zhukov, I.; Fishman, N.; Kiryutin, A.; Lukzen, N.; Panov, M.; Steiner, U.; Vieth, H.-M.; Schäfer, J.; Lambert, C.; Yurkovskaya, A. Positive electronic exchange interaction and predominance of minor triplet channel in CIDNP formation in short lived charge separated states of D-X-A dyads. *J Chem Phys* **2020**, *152* (1), 14203. DOI: 10.1063/1.5131817.

(5) Phillips, J. C.; Hardy, D. J.; Maia, J. D. C.; Stone, J. E.; Ribeiro, J. V.; Bernardi, R. C.; Buch, R.; Fiorin, G.; Hénin, J.; Jiang, W.; McGreevy, R.; Melo, M. C. R.; Radak, B. K.; Skeel, R. D.; Singharoy, A.; Wang, Y.; Roux, B.; Aksimentiev, A.; Luthey-Schulten, Z.; Kalé, L. V.; Schulten, K.; Chipot, C.; Tajkhorshid, E. Scalable molecular dynamics on CPU and GPU architectures with NAMD. *J Chem Phys* **2020**, *153* (4), 44130. DOI: 10.1063/5.0014475.

(6) Phillips, J. C.; Braun, R.; Wang, W.; Gumbart, J.; Tajkhorshid, E.; Villa, E.; Chipot, C.; Skeel, R. D.; Kalé, L.; Schulten, K. Scalable molecular dynamics with NAMD. *J Comput Chem* **2005**, *26* (16), 1781–1802. DOI: 10.1002/jcc.20289.

(7) Pracht, P.; Grimme, S.; Bannwarth, C.; Bohle, F.; Ehlert, S.; Feldmann, G.; Gorges, J.; Müller, M.; Neudecker, T.; Plett, C.; Spicher, S.; Steinbach, P.; Wesołowski, P. A.; Zeller, F. CREST-A program for the exploration of low-energy molecular chemical space. *J Chem Phys* **2024**, *160* (11). DOI: 10.1063/5.0197592.

(8) Neese, F.; Wennmohs, F.; Becker, U.; Riplinger, C. The ORCA quantum chemistry program package. *J Chem Phys* **2020**, *152* (22), 224108. DOI: 10.1063/5.0004608.

(9) Ishii, Y.; Murakami, T.; Ohtori, N. Molecular size and shape effects: Tracer diffusion and the Stokes-Einstein relation. *Journal of Molecular Liquids* **2022**, *346*, 118235. DOI: 10.1016/j.molliq.2021.118235.

(10) Evans, R.; Dal Poggetto, G.; Nilsson, M.; Morris, G. A. Improving the Interpretation of Small Molecule Diffusion Coefficients. *Anal Chem* **2018**, *90* (6), 3987–3994. DOI: 10.1021/acs.analchem.7b05032.

(11) Luchinat, C.; Parigi, G. Collective relaxation of protein protons at very low magnetic field: a new window on protein dynamics and aggregation. *J. Am. Chem. Soc.* **2007**, *129* (5), 1055–1064. DOI: 10.1021/ja0633417.

(12) Charlier, C.; Khan, S. N.; Marquardsen, T.; Pelupessy, P.; Reiss, V.; Sakellariou, D.; Bodenhausen, G.; Engelke, F.; Ferrage, F. Nanosecond time scale motions in proteins revealed by high-resolution NMR relaxometry. *J. Am. Chem. Soc.* **2013**, *135* (49), 18665–18672. DOI: 10.1021/ja409820g.

(13) Kowalewski, J.; Maler, L. *Nuclear Spin Relaxation in Liquids*; CRC Press, 2006. DOI: 10.1201/9781420012194.

(14) Pravdivtsev, A. N.; Yurkovskaya, A. V.; Petrov, P. A.; Ivanov, K. L. A Site-Specific Study of the Magnetic Field-Dependent Proton Spin Relaxation of an Iridium N-Heterocyclic Carbene Complex. *Zeitschrift für Physikalische Chemie* **2017**, *231* (4), 857–865. DOI: 10.1515/zpch-2016-0849.
